# Application of the China Diet Balance Index (DBI-2022) in a Region with a High-Quality Dietary Pattern and Its Association with Hypertension: A Cross-Sectional Study in the Lingnan Population

**DOI:** 10.3390/nu18010043

**Published:** 2025-12-22

**Authors:** Weihua Dong, Jian Wen, Xiaona Zhang, Weiyi Gong, Ping Gan, Panpan Huang, Jiaqi Li, Rongzhen Li, Pengkun Song, Gangqiang Ding

**Affiliations:** 1 National Institute for Nutrition and Health, Chinese Center for Disease Control and Prevention, Beijing 100050, China; dwh19861535785@163.com (W.D.); zhangxn@ninh.chinacdc.cn (X.Z.); gongweiyi@ninh.chinacdc.cn (W.G.); 2 NHC Specialty Laboratory of Food Safety Risk Assessment and Standard Development, Guangdong Provincial Center for Disease Control and Prevention, Guangzhou 511430, China; wenjian19750726@126.com (J.W.); gzgp163@163.com (P.G.); hpp545396861@163.com (P.H.); yc07606@um.edu.mo (R.L.); 3 Key Laboratory of Public Health and Nutrition, National Health Commission of the People’s Republic of China, Beijing 100050, China

**Keywords:** China diet balance index, dietary quality, hypertension, middle-aged and older adults, Lingnan region of China, cross-sectional study

## Abstract

**Background:** The China Diet Balance Index 2022 (DBI-2022), released in 2024, is the latest dietary quality assessment tool developed in alignment with the updated Dietary Guidelines for Chinese Residents (2022). However, its association with hypertension in the Lingnan region—a geographic area distinguished by a unique dietary culture—has not been fully examined. **Objective:** This study aimed to systematically evaluate the dietary quality of Lingnan residents using DBI-2022 and explore its association with hypertension. **Methods:** We analyzed cross-sectional data from the 2015 China Adults Chronic Diseases and Nutrition Surveillance, focusing on 2982 Lingnan residents aged 45 years and older. Dietary information was collected via 3 consecutive days of 24-h dietary recalls and food frequency questionnaires, supplemented by standardized anthropometric measurements. We assessed the contribution of specific dietary components to overall quality and investigated the association between DBI-2022 indices and hypertension using multivariable regression models. **Results:** Among the 2982 participants, 821 (27.5%) were identified with hypertension. The primary dietary imbalances in the Lingnan population were characterized by moderate insufficient consumption (Low Bound Score [LBS] = 40.48) and moderate excessive intake (High Bound Score [HBS] = 22.58), with insufficient intake being the more prominent concern. Cereals, cooking oils, and salt emerged as key contributors to poor dietary quality, whereas soybeans, fruits, adequate water consumption, and dietary diversity were associated with better dietary quality. After adjusting for potential confounders, participants in the highest quartile of Diet Quality Distance (DQD) had significantly higher odds of hypertension (OR = 1.57, 95% CI: 1.05–2.35) compared to those in the lowest quartile. Similarly, the odds were elevated for those with high LBS (OR = 1.88, 95% CI: 1.24–2.87). **Conclusions:** Dietary insufficiency appears to be a more critical issue than excessive consumption among the Lingnan population. Poor dietary quality, particularly insufficient intake of protective foods, is significantly associated with elevated odds of hypertension. These findings support the applicability of DBI-2022 for regional dietary surveillance and highlight key priorities for targeted nutritional intervention strategies.

## 1. Introduction

Hypertension represents a formidable global public health challenge and is a principal modifiable risk factor for numerous severe complications [1,2,3]. In China, the prevalence of hypertension has risen steadily, increasing from 18.8% in 2002 to 27.5% in 2018 [4]. The prevalence exhibits a near-linear increase with age, positioning middle-aged and older adults as a priority population for chronic disease prevention and intervention [5,6].

As a lifestyle-related condition, the pathogenesis of hypertension is intimately linked to diet and nutrition. Targeted interventions based on robust dietary quality assessments have proven to be an effective strategy for mitigating the burden of hypertension [7]; specifically, high-quality diets have been shown to significantly reduce the risk of incident hypertension [8]. Furthermore, improving dietary quality has become a central component of health promotion initiatives. Various dietary quality assessment tools tailored to specific regional dietary structures have been developed globally, including the Healthy Eating Index [9] and the Mediterranean Diet Score [10]. In the Chinese context, researchers established the Diet Balance Index (DBI) based on local dietary characteristics [11]. Compared to earlier iterations, the DBI-2022 incorporates critical revisions following the release of the *Chinese Dietary Guidelines (2022)* [12]. By recalibrating evaluation indicators and scoring systems to align with updated national nutritional benchmarks, the DBI-2022 aims to provide a more precise and scientific reflection of the nutritional status of Chinese residents within the current dietary environment.

The dietary characteristics of regions with high-quality diets often serve as a foundation for developing such assessment tools [13]. The dietary pattern of the Lingnan region, characterized by a diversity of ingredients and rational food combinations, represents a significant component of Southern Chinese dietary culture. This pattern emphasizes the consumption of abundant vegetables and aquatic products, favoring cooking methods that utilize low salt and oil [14,15]. Research indicates that dietary quality in the Jiangnan and Lingnan regions is generally high and associated with a lower burden of chronic diseases [15,16,17,18].

While assessing dietary quality in these regions offers valuable reference points for improvement strategies elsewhere, the utility of specific assessment tools requires verification. The DBI-2022, established based on local Chinese dietary characteristics, is a promising instrument for quantifying dietary quality. However, the validity of the updated DBI-2022 has not yet been established in regions with traditionally high-quality diets. Its previous applications have primarily focused on identifying nutritional deficiencies in regions with low-to-moderate dietary quality [19,20]; consequently, the sensitivity of this index for health risk stratification among populations with traditionally high-quality diets remains largely unverified.

Furthermore, rapid urbanization is driving significant shifts in the dietary structure of Chinese residents. Studies indicate that from 2002 to 2022, the consumption of ultra-processed foods—particularly ready-to-eat products—has steadily increased in the Lingnan region [21]. Concurrently, increased frequency of dining out, excessive consumption of refined staples, and changes in cooking methods may compromise the health benefits of the traditional diet [22,23,24,25]. This trend toward dietary “Westernization” may reduce dietary diversity and the intake of protective nutrients, potentially altering the gut microbiome and increasing the risk of chronic diseases [26,27].

Middle-aged and older adults (aged 45 and above), a population already at high risk for chronic conditions, may be more susceptible to these dietary shifts than younger demographics [28]. This vulnerability may be a key factor driving the increased burden of hypertension in urban areas. Despite this, the quantitative relationship between dietary quality and hypertension in populations residing in high-quality diet regions—specifically among middle-aged and older adults—remains underexplored.

Therefore, research targeting this demographic in the Lingnan context is essential to address current gaps in the literature. This study selected the Lingnan region of China as a representative dietary area. Using a cross-sectional design, we focused on residents aged 45 years and older to assess their dietary quality with the DBI-2022 and to analyze its association with the prevalence of hypertension. The findings of this research are intended to:

(1) Assess the applicability of the DBI-2022 in a region with a traditionally high-quality diet, specifically evaluating its sensitivity in distinguishing health outcomes among populations with high baseline dietary quality.
(2) Quantify dietary quality and examine the relationship between dietary imbalance—encompassing both deficiency and excess—and hypertension.
(3) Identify the specific food components that constitute the strengths and deficits of the Lingnan dietary pattern, thereby providing empirical evidence for the development of regionalized, precision nutrition intervention strategies.

## 2. Materials and Methods

### 2.1. Data Source and Study Population

Data for this study were derived from the 2015 China Adults Chronic Diseases and Nutrition Surveillance. A complex, multi-stage, stratified cluster random sampling method was employed to construct a representative sample covering 31 provinces, autonomous regions, and municipalities in mainland China, comprising a total of 302 randomly selected monitoring sites. The sampling procedure was conducted in four stages:

Stage 1: Three townships or sub-districts were randomly selected from each monitoring site.

Stage 2: Two villages or communities were randomly selected from each sampled township or sub-district.

Stage 3: One village group or residential group containing at least 60 households was randomly selected from each sampled village or community.

Stage 4: Within each monitoring site, 45 households were selected for the general survey, and 20 households were selected for the detailed dietary survey. The sample size of permanent residents aged 18 years and above at each monitoring site was required to be no less than 612 [29].

This study was approved by the Ethics Committee of the Chinese Center for Disease Control and Prevention (Approval No. 201519-B), and informed consent was obtained from all participants prior to their enrollment. This study focuses specifically on the Lingnan region of China (comprising Guangdong, Guangxi, and Hainan provinces), an area characterized by a shared “Lingnan” dietary culture. This shared background ensures a relatively homogeneous dietary baseline among the selected provinces, thereby minimizing regional confounding.

Permanent residents aged 45 years and older were screened for eligibility. Participants were excluded based on the following criteria:

(1) Missing key sociodemographic information (e.g., age, gender, or place of residence);
(2) Incomplete physical examination records, specifically the lack of valid measurements for blood pressure, height, or weight;
(3) Missing data on lifestyle factors (e.g., smoking, alcohol consumption, physical activity) or history of hypertension diagnosis;
(4) Missing or extreme dietary data.

**Figure 1:**
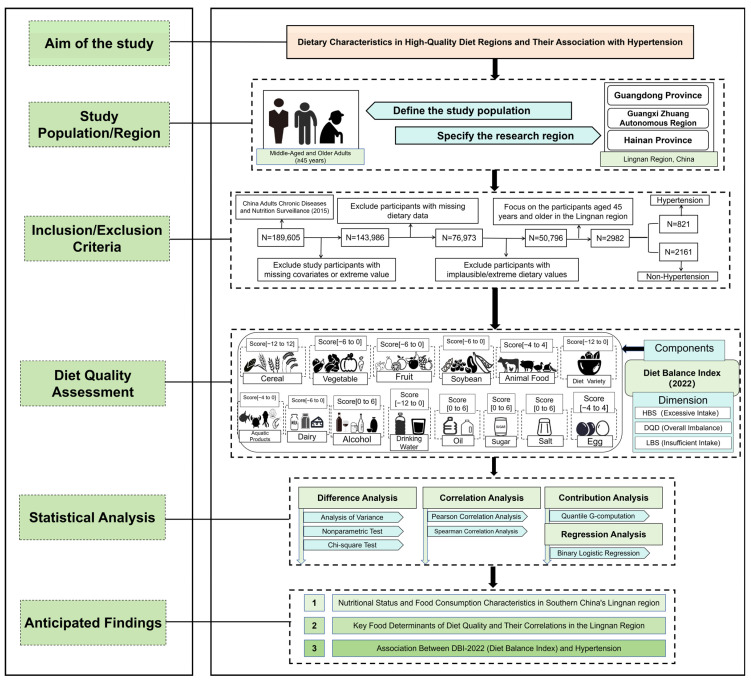
Schematic overview of the study design and participant flow.

Ultimately, a total of 2982 participants were included in the final analysis (Figure 1).

### 2.2. Data Collection and Nutritional Assessment

Trained investigators conducted in-home interviews using standardized questionnaires to collect data on demographics, lifestyle, health status, and physical activity. The dietary assessment comprised several components:

Food-weighting record: Household consumption of edible oil and primary condiments such as salt, sauce, and other seasonings was recorded over a three-day period.

3-day 24-h dietary recall: Participants provided detailed records of all foods, snacks, fruits, and beverages consumed both at home and away from home over three consecutive days (two weekdays and one weekend day).

Food Frequency Questionnaire (FFQ): This tool was used to assess the frequency and portion sizes of specific foods consumed over the past month or year.

Total energy and nutrient intakes were calculated based on data from a 3-day 24-h dietary recall combined with the household food-weighting record. Intake of specific food groups was assessed using a FFQ.

To ensure the accuracy of portion size estimation, trained investigators utilized standard food reference atlases and physical models of tableware (including food scales, standard bowls, cups, and spoons) to assist participants in quantifying their intake. The consumption of cooking oil, salt, and other condiments was assessed by recording the precise changes in household inventory weights over three consecutive days. Individual intake was subsequently apportioned based on the ratio of the individual’s energy intake to the total energy consumption of all household members dining at home during the survey period.

Anthropometric measurements were conducted by certified technicians using standardized equipment (TZG stadiometer, Wuxi, China, and Tanita HD-390 digital scale, Dongguan, China). Height (to the nearest 0.1 cm), weight (0.1 kg), and blood pressure were measured following standard protocols, with participants wearing light clothing and no shoes. Blood pressure was measured three times, and the mean of the three readings was used for the final analysis.

Physical activity assessment focused specifically on Leisure-Time Physical Activity, defined here as voluntary health-promoting behaviors. Participants self-reported the frequency and duration of activities performed specifically for exercise or recreation. Total physical activity duration was calculated based on these reports. Activity intensity was classified according to physiological response:

(1) Vigorous Intensity: Activities that cause a significant increase in breathing or heart rate and are sustained for at least 10 min.
(2) Moderate Intensity: Activities that cause a mild increase in breathing or heart rate and are sustained for at least 10 min.

### 2.3. Definition of Hypertension

Participants were classified as having hypertension if they met one or more of the following criteria: (1) a measured systolic blood pressure ≥140 mmHg and/or diastolic blood pressure ≥90 mmHg; (2) current use of antihypertensive medication; (3) a self-reported physician diagnosis of hypertension; or (4) self-reported adoption of lifestyle modifications specifically aimed at controlling blood pressure (e.g., dietary adjustments or physical activity). The inclusion of the lifestyle modification criterion ensures the capture of participants who may be effectively managing a diagnosed condition through therapeutic behavioral changes in the absence of pharmacological intervention. The 140/90 mmHg threshold was based on the 2018 Chinese Guidelines for the Management of Hypertension [30].

### 2.4. Dietary Quality Assessment

Dietary quality was evaluated using the revised China Diet Balance Index (DBI-2022). The DBI-2022 is a comprehensive assessment tool based on the *Chinese Dietary Guidelines (2022)* [12]. Its components include cereals, vegetables, fruits, dairy products, soybeans, animal-source foods, high-energy foods, condiments, dietary diversity, and water intake. The index concurrently reflects both insufficient and excessive dietary intake through three primary scores: the High Bound Score (HBS) for overconsumption, the Low Bound Score (LBS) for underconsumption, and the Diet Quality Distance (DQD) score, which represents the overall dietary imbalance. For all three scores, a higher value indicates poorer dietary quality. Each score is also categorized into five levels: Good, Acceptable, Mild, Moderate, and Severe. The specific scoring methodology is detailed in References [31,32] and Supplementary Tables S1 and S2.

### 2.5. Covariates

Sociodemographic factors: Age (45–59, 60–74, or ≥75 years), ethnicity (Han or other ethnic groups), education level (primary school or below, secondary/high school, or college or above), residence (urban or rural), marital status (married/cohabiting/separated or unmarried/widowed/divorced), and monthly income (<5000, 5000–9999, or ≥10,000 CNY).

Lifestyle factors: Smoking status (daily smoker or occasional smoker), alcohol consumption (≥1 drink in the past year), and adequate physical activity (≥150 min of moderate-intensity or ≥75 min of vigorous-intensity activity per week) [33].

Physiological factors: Body Mass Index (BMI), categorized as underweight (<18.5 kg/m^2^), normal weight (18.5–23.9 kg/m^2^), overweight (24.0–27.9 kg/m^2^), or obese (≥28.0 kg/m^2^) [34]; family history of chronic disease (yes/no); multimorbidity was defined as the concurrent presence of two or more chronic conditions, specifically hypertension, type 2 diabetes, and dyslipidemia; polypharmacy was defined as the concurrent use of two or more medications for these specific conditions (including antihypertensives, hypoglycemic agents, or lipid-lowering drugs).

### 2.6. Statistical Analysis

Categorical variables were presented as counts and percentages (n, %), with between-group differences assessed using the chi-square test. Normally distributed continuous variables were reported as mean (standard deviation) and compared using analysis of variance (ANOVA). Non-normally distributed continuous variables were described as median (25th, 75th percentile) and compared using non-parametric tests. The Mantel-Haenszel test was used for trend analysis of ordered categorical variables. Pearson or Spearman correlation coefficients were calculated to assess the relationships among dietary components and with the DBI-2022 scores.

A Quantile g-Computation (Qgcomp) model was employed to evaluate the contribution of each dietary component to the overall DBI-2022 score, elucidating the positive or negative weight of each component. Qgcomp is a statistical method for estimating the joint effect of a mixture of exposures and was implemented using the qgcomp package in R software (version 4.5.1). This method does not require a priori assumptions that all exposures affect the outcome in the same direction and accommodates non-linear relationships. The Qgcomp model relies on two core assumptions: first, standard causal identification (i.e., consistent individual response to exposure); and second, correct model specification (i.e., the model structure accurately captures complex relationships, such as non-linearity or non-additivity). Although the model has limitations in capturing high-dimensional complex interactions, it offers a superior balance for interpretability given the study’s objective to clearly quantify the independent contribution weights of dietary components. Furthermore, the robustness of the estimates is supported by the study’s large sample size and strict exclusion criteria, which meet the model’s data quality requirements [35]. The model is described as follows:

$$Y_{i}=\beta_{0}+\sum_{j=1}^{k}\beta_{i}X_{qji}+\varepsilon_{i}$$

where $Y_{i}$ represents the total DBI-2022 score for individual i; $X_{qji}$ represents the quantized intake level of the j-th food group (e.g., grains, vegetables, dairy); $\beta_{i}$ is the coefficient for the j-th food group, indicating the magnitude and direction of its contribution to the final DBI score; and $\varepsilon_{i}$ is the error term.

In this study, the Qgcomp model bootstrap iterations were set to 10,000, and the parameter q was set to 4. To evaluate goodness-of-fit, the coefficient of determination (*R*^2^) from a generalized linear model was calculated to determine the proportion of variance in the outcome explained by the dietary mixture exposure.

Multivariable binary logistic regression models were used to analyze the association between DBI-2022 scores and hypertension. The likelihood ratio test was further employed to explore these associations and potential interaction effects within various subgroups. To ensure the robustness of the findings, sensitivity analyses were also performed, which involved re-running the models under several predefined scenarios(Table 1):

Three logistic regression models were constructed with stepwise adjustments for potential confounders:

(1) Model 1: Unadjusted.
(2) Model 2: Adjusted for sociodemographic characteristics, including gender, age, marital status, residence, education level, ethnicity, and household income.
(3) Model 3 (Fully Adjusted Model): Further adjusted for lifestyle and health-related factors, including family history of chronic diseases, BMI category, physical activity, smoking status, alcohol consumption, multimorbidity, and polypharmacy.

Age and BMI were entered as categorical rather than continuous variables to: (1) maintain consistency with stratified subgroup analyses; and (2) adequately control for the confounding effects without assuming a strict linear relationship with hypertension. The Variance Inflation Factor (VIF) was calculated to examine potential multicollinearity among covariates. A cut-off value of 5 was applied, where a VIF < 5 indicated acceptable independence between variables.

Participants with missing data for any covariates were excluded to maintain analytical rigor. However, given the large sample size, this exclusion is unlikely to compromise the generalizability of the findings. All statistical analyses were conducted using R software (version 4.5.1) and SAS software (version 9.4; SAS Institute Inc., Cary, NC, USA). A two-sided *p*-value < 0.05 was considered statistically significant.

## 3. Results

### 3.1. Characteristics of Participants

This study included 2982 residents aged 45 years and older from the Lingnan region, of whom 821 (27.53%) were classified as having hypertension. Significant differences were observed between the hypertension and non-hypertension groups with respect to gender, age, residence, income, family history of chronic diseases, BMI, physical activity, multimorbidity, and polypharmacy (all *p* < 0.05).

The hypertension group had a higher proportion of males (423 [51.52%] vs. 990 [45.81%]), was older (median age 61.70 vs. 57.61 years), included more urban residents (355 [43.24%] vs. 822 [38.04%]). Additionally, BMI levels were significantly higher in the hypertensive group (median 24.46 vs. 22.68 kg/m^2^), with a higher number of obese participants (119 [14.49%] vs. 118 [5.46%]).

Furthermore, the proportions of individuals with a family history of chronic diseases, multimorbidity, and polypharmacy were significantly higher in the hypertension group (all *p* < 0.001). However, there were no significant differences in the mean HBS, LBS, and DQD scores between the two groups (Table 2).

### 3.2. Distribution of Participants Across DBI-2022 Categories

**Figure 2:**
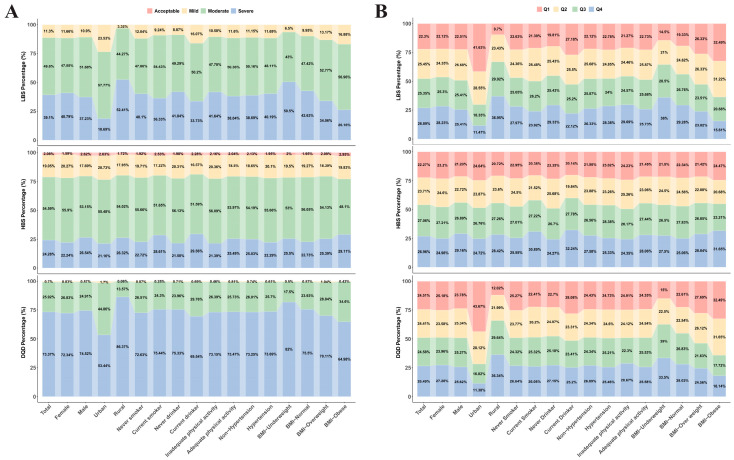
Distribution of participants by dietary quality categories and score quartiles of the DBI-2022. (**A**) Proportions of subgroups across distinct DBI dimensions; (**B**) Proportions of subgroups categorized according to DBI quartiles. Detailed descriptions of the data visualization and chart elements are provided in the Supplementary Text.

The classification of dietary quality revealed that no participant achieved a ‘Good’ rating. Within the overall population, the ‘Severe’ imbalance group of the DQD accounted for the highest proportion (73.37%). Significant disparities were observed regarding gender and place of residence. Males constituted a significantly higher proportion of the “Severe” group for the HBS compared to females (51.80% vs. 48.20%, *p* = 0.005). Notably, rural residents were disproportionately represented in the “Severe” categories for DQD, HBS, and LBS compared to urban residents (DQD: 71.25% vs. 28.75%; HBS: 65.61% vs. 34.39%; LBS: 81.13% vs. 18.87%; all *p* < 0.01). However, the distribution across these categories did not differ significantly between the hypertension and non-hypertension groups (Figure 2A, Table S3).

Analysis of the quartile distribution for DBI-2022 components indicated distinct demographic clustering patterns. Females were predominantly concentrated in the highest quartile (Q4) for DQD and LBS (accounting for approximately 54–55%), whereas males exhibited a higher prevalence in the highest quartile for HBS. Urban-rural disparities were particularly pronounced, with rural residents accounting for a substantially higher proportion of the highest quartiles across all three dimensions (DQD, HBS, and LBS; range: 63–83%, *p* < 0.05). No significant differences were observed across quartiles with respect to hypertension status (*p* > 0.05) (Figure 2B, Table S4).

### 3.3. DBI-2022 Scores and Dietary Intake Characteristics

In the overall study population, the mean scores for the DQD, HBS, and LBS were 63.06, 22.58, and 40.48, respectively. Subgroup analyses revealed that urban-rural disparities were the most pronounced. Rural residents scored significantly higher across all three dimensions (DQD, HBS, and LBS) compared to urban residents (*p* < 0.01).

**Figure 3:**
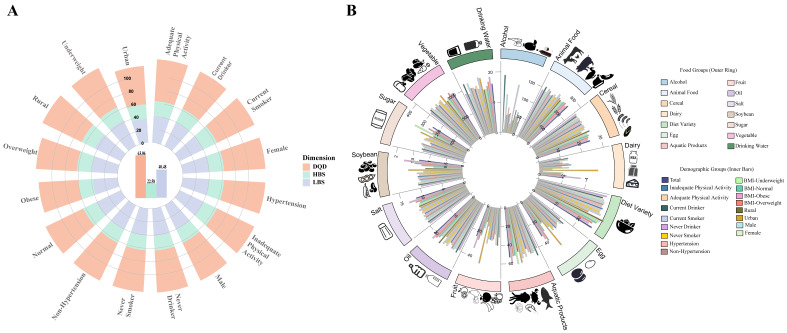
Mean DBI-2022 scores and dietary component intake across population subgroups. (**A**) DBI dimension scores in the total population and different subgroups. (**B**) Intake of DBI food components in the total population and different subgroups. Detailed descriptions of the data visualization and chart elements are provided in the Supplementary Text.

In contrast, gender and lifestyle factors exhibited statistically significant yet smaller magnitudes of difference, while presenting consistent behavioral patterns. Males demonstrated slightly higher scores for dietary excess (HBS) compared to females. Similarly, HBS scores were marginally higher among smokers and alcohol consumers compared to non-smokers and non-drinkers (Figure 3A, Table S5).

Analysis of food intake revealed distinct demographic and behavioral patterns (Table S4). Females tended to consume higher quantities of plant-based foods (vegetables and fruits) and dairy products, whereas males consumed more animal-based foods and alcohol. Urban-rural comparisons indicated a more diverse dietary structure among urban residents, characterized by significantly higher consumption of vegetables, fruits, dairy products, aquatic products, eggs, and legumes. Conversely, rural residents reported a greater proportional intake of cereals and cooking oils, accompanied by higher alcohol consumption (*p* < 0.001).

Furthermore, lifestyle factors and body weight status were closely associated with specific food choices. Smokers exhibited a dietary pattern characterized by “low vegetable/fruit and high meat” consumption, alongside elevated alcohol intake. Alcohol consumers were found to consume higher quantities of aquatic products and eggs. Regarding BMI stratification, overweight and obese individuals generally reported higher overall food intake, including vegetables, fruits, dairy products, and salt. Notably, dietary diversity scores were superior among urban residents and non-smokers (Figure 3B, Table S6).

### 3.4. Contribution of Dietary Components to DBI-2022 Scores

**Figure 4:**
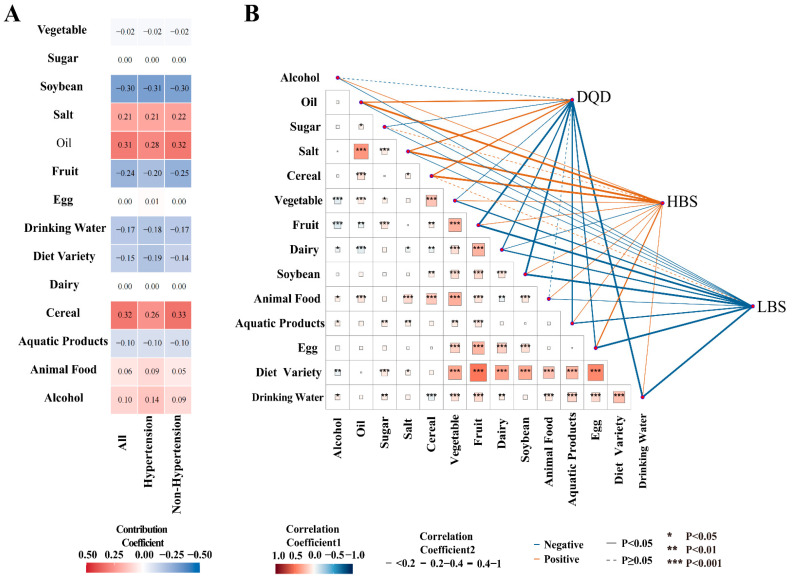
Contribution of dietary components to overall dietary imbalance and their intercorrelations. (**A**): Contribution of various food components to the DBI score; (**B**): Correlations between DBI dimensions and food components, and inter-correlations among food components. Detailed descriptions of the data visualization and chart elements are provided in the Supplementary Text.

Given that the DQD score provides a comprehensive measure of overall dietary imbalance by integrating all dietary components, we used it as the primary outcome in our Qgcomp model to assess the influence of each component on dietary quality. The results were consistent across the total population and when stratified by hypertension status. Cereals, oil, and salt were the top three contributors to poorer dietary quality (i.e., higher DQD scores), exhibiting the strongest positive weights. Alcohol and animal-source foods also demonstrated notable positive contributions. Conversely, soybeans, fruits, and dietary diversity were the top three factors associated with better dietary quality, showing strong negative weights. Water intake and aquatic products also played a significant role in improving dietary quality (Figure 4A). Furthermore, the combined exposure to all food components explained between 75% and 77% of the variance in overall dietary imbalance (R^2^ = 0.7672 for the total population; R^2^ = 0.7685 for the non-hypertensive group; and R^2^ = 0.7579 for the hypertensive group).

### 3.5. Correlation Analysis

A significant positive correlation was observed between oil and salt intake. Dietary diversity was positively correlated with the consumption of fruits, vegetables, dairy products, soybeans, animal-source foods, aquatic products, and eggs.

The DQD score was positively correlated with the intake of cereals, oil, and salt, and negatively correlated with the intake of vegetables, fruits, dairy products, soybeans, aquatic products, eggs, sugar, water, and dietary diversity. The HBS was positively correlated with the consumption of cereals, animal-source foods, eggs, oils, alcohol, sugar, and salt. The LBS was negatively correlated with the consumption of vegetables, fruits, dairy products, soybeans, animal-source foods, aquatic products, eggs, water, and dietary diversity (Figure 4B, Table S7).

### 3.6. Association Between DBI-2022 and Hypertension

After full adjustment for covariates (Model 3), logistic regression analysis revealed a significant positive association between poorer dietary quality and hypertension. Compared with participants in the lowest DQD quartile (Q1), those in the highest quartile (Q4) had a 57% higher odds of hypertension (OR = 1.57, 95% CI: 1.05–2.35). Subgroup analysis indicated this association was more pronounced in men, never-smokers, drinkers, physically active individuals, and those who were overweight. A significant interaction was detected between DQD and physical activity (*p* for interaction = 0.01).

A positive association between the LBS and hypertension was also observed. Participants in the highest LBS quartile (Q4) had an 88% greater odds of hypertension compared with those in the lowest quartile (OR = 1.88, 95% CI: 1.24–2.87). This association was stronger among men, rural residents, smokers, drinkers, physically active individuals, and those with a normal BMI. There was a significant interaction between LBS and alcohol consumption (*p* for interaction = 0.03).

**Figure 5:**
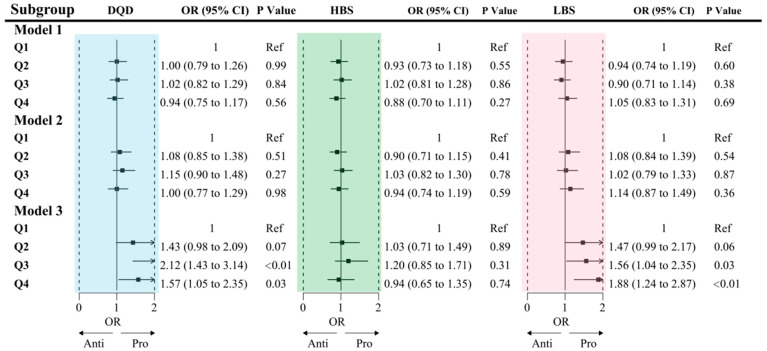
Association between quartiles of dietary quality scores and odds of hypertension.

**Figure 6:**
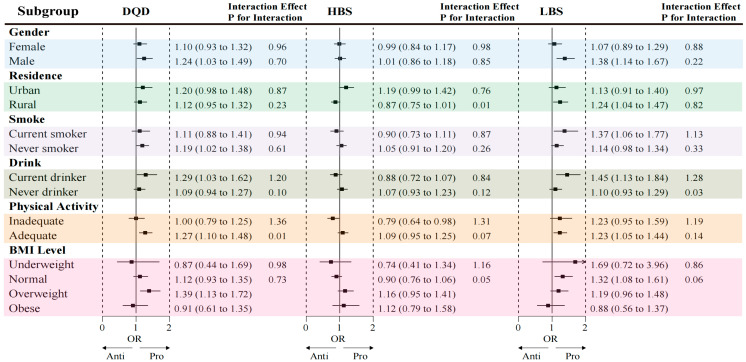
Subgroup analysis of the association between quartiles of dietary quality scores and odds of hypertension. Detailed descriptions of the data visualization and chart elements are provided in the Supplementary Text.

Furthermore, a significant interaction was found between the HBS and residence (P for interaction = 0.01), with the positive association between excessive intake and hypertension being evident only among urban residents (Figure 5 and Figure 6, Table S8).

We also conducted sensitivity analyses to assess the robustness of our findings. Across all scenarios examined, we observed no substantive changes in the magnitude of the association between DBI-2022 scores and the odds of hypertension, indicating that this significant association is robust (Table S9).

## 4. Discussion

### 4.1. Prevalence and Correlates of Dietary Imbalance in Lingnan

The Lingnan diet has long been recognized for its traditional emphasis on “light and fresh” characteristics. By identifying the key dietary components that likely contribute to these benefits, this study offers valuable reference points for regional dietary guidance. However, the findings reveal a potential paradox: despite economic prosperity and an abundance of food, this “ideal diet” appears to be diminishing within the general population. Given that no participant achieved a “Good” standard as defined by the DBI-2022, our results suggest that the primary dietary challenge in the region is no longer merely “overt surplus” (i.e., surplus oil, salt, and sugar), but rather “latent deficiency”—a systemic deficiency in protective foods.

Our analysis indicates that the key factor contributing to this low dietary quality is not simply a caloric surplus, but a severe insufficiency of key nutrient carriers, particularly fruits, dairy, and soy products. This finding highlights the unique nature of the nutritional transition in Southern China: economic growth has not effectively translated into optimized dietary structures but has instead been accompanied by a contradiction between high energy density and low nutrient density. This conclusion aligns with previous findings from Wenzhou, Guangzhou, and Beijing [19,20,36,37,38], suggesting that the “latent deficiency” of healthy foods has evolved into a deep-seated public health challenge across regions.

Although China is characterized by significant regional dietary diversity, the emergence of similar dietary quality issues across different provinces reflects fundamental deficiencies in the recent transformation of the national dietary structure. These include reduced whole grain intake, increasingly high consumption of animal-based foods, and a substantial “Westernization” of dietary patterns [25,39,40,41], which have coincided with an escalating burden of chronic diseases [42]. Epidemiological data indicate that overweight and obesity rates among Chinese adults are rising [43] and are closely associated with the high prevalence of cardiovascular diseases and diabetes [44,45].

This regional convergence of dietary issues may be influenced by two primary factors. First, the standardization of market supply has reduced the spatial heterogeneity of food availability, potentially contributing to a homogenization of consumption patterns [46,47]. Second, rapid urbanization has reshaped lifestyles; the proliferation of fast-food culture and the increase in high-density social dining services have significantly reduced the time and choice costs for consumers, further reinforcing dietary uniformity [48,49]. These trends suggest that modern supply chains and shifting eating habits may be gradually compromising traditional regional dietary advantages.

### 4.2. Association Between Dietary Quality and Hypertension

Dietary imbalance is strongly associated with an elevated likelihood of disease. In the current study, although unadjusted comparisons revealed no statistically significant differences in mean DBI scores between hypertensive and non-hypertensive groups, these raw associations were likely obscured by confounding factors. Upon adjustment for potential confounders, poor dietary quality exhibited a significant positive association with hypertension. Specifically, participants in the highest quartile (Q4) of the DQD had 57% higher odds of hypertension (OR = 1.57; 95% CI: 1.05–2.35) compared to the lowest quartile. Furthermore, the odds were elevated by 88% for those in the highest quartile of the LBS (OR = 1.88; 95% CI: 1.24–2.87).

These findings corroborate previous research utilizing the DBI. Elevated LBS have previously been significantly associated with a 4.9-fold increase in the hazard of diabetes (HR: 4.90; 95% CI: 2.46–9.78) [50] and a 2.58-fold increase in the odds of sarcopenia (OR: 2.58; 95% CI: 1.13–7.04) [51]. Similarly, high DQD scores have been linked to ischemic stroke (HR: 2.26; 95% CI: 1.28–4.00) [52] and dyslipidemia [53]. A high LBS signifies a severe deficiency in healthy foods such as fruits, vegetables, and soy, reflecting a reduced intake of protective nutrients, which may contribute to increased physiological susceptibility to elevated blood pressure [54,55]. Indeed, prior literature indicates that, aside from high sodium intake, the primary dietary factors associated with non-communicable diseases are characterized by an insufficient intake of protective foods (e.g., whole grains, fruits, and nuts) rather than solely by an excessive intake of unhealthy components (e.g., fats and sugars) [56].

The results of this study further extend this chain of evidence, providing robust support for the hypothesis that within the Chinese population, “underconsumption of protective foods”—rather than “overconsumption” alone—likely represents a central dietary factor associated with a spectrum of metabolic disorders, including hypertension, diabetes, and sarcopenia. This suggests that future intervention strategies may need to pivot from the traditional paradigm of “limiting excess” to an equally critical focus on “addressing insufficiency” and improving overall dietary balance.

### 4.3. Interaction of Lifestyle and Urbanization

Subgroup analysis suggested a potential “clustering tendency” of maladaptive health behaviors. Although score differences observed within lifestyle subgroups were small in magnitude, they remained statistically significant and hold public health relevance, hinting at the complex interplay of multiple factors. This interplay was subsequently further explored in the association analysis, which demonstrated that the relationship between dietary quality and hypertension is modified by lifestyle and environmental factors.

First, regarding the interaction between overall dietary quality (DQD) and physical activity: contrary to intuition, the association between poor diet and hypertension was more pronounced among participants reporting adequate physical activity. This phenomenon may be interpreted through both epidemiological and behavioral lenses. Epidemiologically, unhealthy diets often cluster with other maladaptive lifestyles, such as smoking and sedentary behavior [57,58,59]. This “clustering” may generate a metabolic “saturation effect,” wherein the specific association of a poor diet is attenuated or masked by the combined presence of multiple co-existing adverse factors [60]. This underscores the difficulty of independently evaluating single factors when multiple adverse behaviors coexist [57]. Behaviorally, we hypothesize that this finding may be attributed to “Compensatory Health Beliefs,” where active individuals rationalize the intake of high-energy or high-sodium foods as a reward for exercise [61,62]. Consequently, the potential protective association of physical activity may be inadvertently offset by these post-exercise dietary choices.

Second, regarding the interaction between dietary insufficiency (LBS) and alcohol consumption: the higher odds of hypertension observed among alcohol consumers with high LBS suggest a potential biological synergistic link. Long-term alcohol consumption may exacerbate sodium-induced fluid retention by enhancing salt sensitivity; when combined with other adverse lifestyle factors—including dietary deficits—this interaction is linked to significantly higher odds of hypertension [63,64,65,66,67].

Third, regarding urbanization: the association between dietary excess (HBS) and hypertension was significantly stronger in urban populations. This finding likely reflects structural differences in the sources of the DBI’s “excess” components (e.g., cooking oil, sodium, and animal-based foods). Although urban areas typically offer superior healthcare access [68], the “environmental factors” of urbanization—such as the high accessibility of ultra-processed foods, sedentary lifestyles, and environmental pollution—may compound the metabolic burden associated with dietary excess [68,69].

These paradoxes indicate that pervasive dietary issues persist even in regions with relatively higher dietary quality, manifesting as distinct patterns of association within specific subgroups. This necessitates a deeper examination of the sociodemographic and behavioral factors underlying suboptimal diets and highlights the necessity of a stratified intervention paradigm.

For instance, the elevated likelihood of hypertension observed in physically active individuals with poor diets exposes a specific cognitive misalignment—likely driven by Compensatory Health Beliefs—that warrants targeted education to correct erroneous beliefs regarding post-exercise dietary indulgence. For populations where alcohol cessation proves challenging, optimizing the intake of protective foods could be considered a strategy to potentially mitigate alcohol-related vascular issues. While caution is required in interpreting these results due to potential under-reporting bias in self-reported alcohol data, the implication regarding the critical role of nutritional improvement in disease control remains clear. Conversely, urban-rural disparities reflect structural differences in the food environment; therefore, targeted environmental modification strategies should be prioritized during urbanization. These include strengthening the regulation and nutritional labeling of catering services and ultra-processed foods to address metabolic burdens at the source. Therefore, the formulation of dietary assessment metrics and public health policies would benefit from extending beyond the biological health effects of nutrients to also incorporate the accessibility, affordability, and behavioral contexts of diverse populations.

### 4.4. Key Dietary Indicators and Potential Biological Mechanisms

In our study, soybeans, fruits, water, dietary diversity, and aquatic products were identified as “key dietary indicators” for the Lingnan region. These components showed strong negative contributions in the Qgcomp model and were inversely associated with poor diet scores. Although the absolute intake of fruits, soy products, and water in the study population did not fully meet the recommended standards of the Dietary Guidelines for Chinese Residents [12], these components displayed high weights in the mixed exposure effect analysis using the Qgcomp model, significantly correlating with better DBI-2022 scores. This suggests that within the overall dietary pattern of the Lingnan region, these indicators constitute the relatively healthy components.

The Lingnan region is abundant in fruit, vegetable, and aquatic resources [14], and these food groups have been previously linked to positive health outcomes regarding chronic diseases such as hypertension [70,71,72,73]. Plant-based foods, characterized by low energy density and high fiber, potassium, and magnesium content, are thought to support improved baroreflex sensitivity and endothelium-dependent vasodilation [54]. Furthermore, bioactive compounds such as soy isoflavones have been shown to be associated with improved endothelial function and nitric oxide bioavailability, thereby suggesting potential antihypertensive mechanisms [55]. Additionally, the association observed with water intake may be attributable to the long-standing tea-drinking tradition in the Lingnan region; phytochemicals in tea possess anti-inflammatory, antioxidant, lipid-lowering, and blood pressure-lowering properties, as well as beneficial effects on the gut microbiota [74,75,76,77].

The high-weight indicators identified by the Qgcomp model provide distinct targets for regional precision nutrition interventions. Specifically, in the context of Lingnan dietary culture, intervention strategies need to extend beyond generic advice for a balanced diet. Instead, strategies should leverage existing dietary advantages by reinforcing the intake of plant-based foods and aquatic products to optimize the potential health benefits of these protective components.

Our study did not account for cooking methods, which may be a limitation. Lingnan cuisine emphasizes steaming and stir-frying over deep-frying [14]. These methods better preserve nutrients and avoid the formation of harmful compounds associated with high-heat frying [78,79], the overconsumption of which is linked to cardiovascular and metabolic diseases [80,81]. Therefore, the promotion of the Lingnan diet should also emphasize its healthful cooking philosophy.

### 4.5. The Inverse Association of Refined Cereals, Oil, and Salt with Dietary Quality

Furthermore, the Qgcomp model analysis indicated that high intake of cooking oil, salt, and cereals constituted factors contributing to lower dietary quality in the Lingnan region. Although the adverse health associations of excessive oil and salt consumption are well-established [82,83], the negative weight assigned to cereals warrants cautious interpretation, necessitating a clear distinction between the potential health implications of refined grains and whole grains.

The inverse association observed for cereals in this study may be attributed to their predominant role in the traditional Chinese dietary structure and their highly processed nature. Data from 2015 indicate that carbohydrates provide over 50% of dietary energy for the Chinese population, derived primarily from cereals [84]. Crucially, the staple diet in Southern China consists mainly of refined grains, such as white rice [85]. Unlike whole grains, refined grains are stripped of substantial dietary fiber and micronutrients during milling and possess a higher glycemic index [86]. Previous studies have indicated that excessive intake of refined grains is associated with an increased risk of cardiovascular disease, whereas whole grain intake has been linked to protective effects [87]. Therefore, the negative contribution of cereals identified in this study likely reflects a structural imbalance characterized by an overconsumption of refined grains and an insufficiency of whole grains. Consequently, when formulating dietary recommendations based on Lingnan dietary characteristics, strategies could not simply advocate for “reducing cereals,” but rather explicitly emphasize “substituting refined grains with whole grains” to address this specific qualitative imbalance.

### 4.6. Suggestions for Regional Nutritional Strategies

The analysis of the aforementioned key indicators and factors associated with lower diet quality offers a basis for regional nutritional improvement:

First, programs could implement precise education focused on “Whole Grain Substitution” and “Oil and Salt Reduction.” These programs would benefit from promoting the concept of combining whole grains with refined grains, encouraging the addition of brown rice, oats, or mixed legumes to staple rice dishes.

Second, integrating traditional culinary techniques into health promotion initiatives is recommended. It is important to preserve and promote traditional Lingnan cooking methods such as steaming, blanching, and rapid stir-frying. These low-temperature, short-duration cooking methods may serve as valuable strategies to mitigate modern culture’s reliance on high-calorie processed foods. Emphasizing a “light diet” could be framed not merely as a tradition, but as a scientifically grounded lifestyle approach for hypertension management.

Finally, efforts to enhance the accessibility and affordability of protective foods are warranted. To address the insufficiency of protective food intake, policy recommendations might focus on optimizing supply chains to increase the availability of fruits and soy products in community markets and workplace canteens. Furthermore, leveraging the Lingnan tea culture, “tea drinking” could be promoted as a way to increase water intake to help residents meet recommended daily water intake standards.

### 4.7. Strengths and Limitations

The primary strength of this study lies in its dual innovation. First, this study represents the first systematic application and validation of the latest DBI-2022 within the Lingnan population, a group characterized by a traditionally high-quality diet. This not only confirms the sensitivity and applicability of the index across different dietary cultural contexts but also provides empirical support for the standardized application of dietary quality indices across regions. Second, the study quantified the association between dietary quality and hypertension, revealing that “dietary insufficiency” rather than “excess” is the predominant feature of dietary imbalance in this region. This critical finding provides a more targeted direction for future intervention strategies: prioritizing the increased intake of healthy foods rather than solely focusing on controlling excessive consumption.

However, several limitations warrant consideration. First, the cross-sectional design precludes the inference of causal relationships; findings are limited to associations. Future research should consider long-term prospective cohort studies with repeated dietary measurements rather than a single baseline assessment to elucidate the causal link between dietary quality and disease incidence.

Second, although multiple covariates were adjusted for, the possibility of residual confounding due to unmeasured factors remains. The relatively wide confidence intervals observed in the logistic regression suggest a degree of estimation uncertainty. This variation may stem from unmeasured determinants of hypertension, such as genetic factors. This suggests that future studies should incorporate genomic characteristics or more granular environmental exposure data to further mitigate residual confounding and improve the precision of effect estimates.

Furthermore, as a food-group-based composite index, the DBI-2022 may not precisely capture the intake of all micronutrients, particularly those closely linked to the pathophysiology of hypertension. Future research should aim to integrate index-based assessments with specific micronutrient analyses to generate a more comprehensive profile of diet-disease associations.

Finally, given the relative reduction in sample size following subgroup stratification, the estimates may lack precision; therefore, the observed interactions should be interpreted as exploratory. While these findings provide preliminary clues for identifying high-risk subgroups, they require validation in independent cohorts with larger sample sizes.

Despite these limitations, this study provides valuable regional evidence for understanding the relationship between high-quality Chinese dietary patterns and chronic disease risk, laying a foundation for the development of more precise nutritional improvement strategies.

## 5. Conclusions

The population in the Lingnan region exhibits a degree of dietary imbalance characterized primarily by the underconsumption of healthy foods. This suggests that the long-term scarcity of protective foods may be a significant factor contributing to the region’s overall low dietary quality. Concurrently, the significant association observed between poor dietary quality and hypertension supports the potential value of dietary structural improvement in chronic disease management and control.

Additionally, plant-based foods, aquatic products, adequate water intake, and dietary diversity were identified as core components of a high-quality Lingnan diet, reflecting the health potential of the regional dietary culture. However, the tendency toward high oil and salt consumption appears to be counteracting this advantage. Regarding cereals, a distinction should be made between sources; it is recommended that future dietary guidelines refine recommendations by explicitly differentiating between refined and whole grains, encouraging the substitution of whole grains while noting the potential adverse associations of excessive refined grain intake.

Based on current findings, future public health interventions would benefit from prioritizing the intake of plant-based foods and aquatic products to optimize protective nutrient levels, alongside the promotion of educational programs focused on healthy cooking methods. Overall, although limited by the cross-sectional design and potential bias inherent in self-reported data, the results support the applicability of the DBI-2022 in this regional population and provide empirical insights for understanding the regional characteristics of dietary quality, thereby informing the formulation of more targeted nutritional improvement strategies.

## Figures and Tables

**Table 1:** Description of Sensitivity Analysis Scenarios and Detailed Exclusion Criteria.

Sensitivity Analysis Scenario	Detailed Exclusion Criteria
Scenario 1: Exclusion of Prior Diagnosis	Exclude individuals with a prior diagnosis of hypertension.
Scenario 2: Exclusion of Medication Users	Exclude individuals with a prior diagnosis of hypertension who are currently using antihypertensive medications for blood pressure control.
Scenario 3: Exclusion of Lifestyle Modifications	Exclude individuals with a prior diagnosis of hypertension who are implementing non-pharmacological measures for hypertension control.
Scenario 4: Exclusion of Any Hypertension Control	Exclude individuals with a prior diagnosis of hypertension who are implementing any form of hypertension control measures.
Scenario 5: Exclusion of Control Behaviors	Exclude only those individuals who are implementing any form of hypertension control measures (regardless of diagnosis status).
Scenario 6: Exclusion of Other Interventions	Exclude individuals who are implementing dietary or exercise interventions specifically for the management of other chronic conditions.
Scenario 7: Exclusion of Lifestyle-Only Management	Exclude individuals who are managing hypertension exclusively through lifestyle modifications without the use of antihypertensive medication.

**Table 2:** Characteristics of the study participants.

	ALL n = 2982	Non-Hypertension n = 2161	Hypertension n = 821	Test Statistic	*p* Value
Gender [n (%)]				7.554	0.006
Female	1569 (52.62%)	1171 (54.19%)	398 (48.48%)		
Male	1413 (47.38%)	990 (45.81%)	423 (51.52%)		
Age(years) (M,P25,P75)	58.58 [51.56;66.16]	57.61 [50.95;64.94]	61.70 [53.84;68.81]	−8.742	<0.001
Age Group [n (%)]					<0.001
45–59 years	1628 (54.59%)	1270 (58.77%)	358 (43.61%)	58.723	
60–74 years	1097 (36.79%)	734 (33.97%)	363 (44.21%)		
≥75 years	257 (8.62%)	157 (7.27%)	100 (12.18%)		
Ethnicity [n (%)]				2.444	0.118
Han Chinese	2445 (81.99%)	1787 (82.69%)	658 (80.15%)		
Other ethnic groups	537 (18.01%)	374 (17.31%)	163 (19.85%)		
Residence [n (%)]				6.523	0.011
Urban	1177 (39.47%)	822 (38.04%)	355 (43.24%)		
Rural	1805 (60.53%)	1339 (61.96%)	466 (56.76%)		
Education level [n (%)]				2.411	0.300
Primary school or below	1678 (56.27%)	1225 (56.69%)	453 (55.18%)		
Secondary or high school	1201 (40.27%)	868 (40.17%)	333 (40.56%)		
College or above	103 (3.45%)	68 (3.15%)	35 (4.26%)		
Marital status [n (%)]				0.829	0.362
Unmarried, widowed, divorced	210 (7.04%)	146 (6.76%)	64 (7.80%)		
Married, cohabiting, separated	2772 (92.96%)	2015 (93.24%)	757 (92.20%)		
Monthly income [n (%)]				8.901	0.012
<5000 CNY/month	2241 (75.15%)	1655 (76.58%)	586 (71.38%)		
5000–9999 CNY/month	540 (18.11%)	366 (16.94%)	174 (21.19%)		
≥10,000 CNY/month	201 (6.74%)	140 (6.48%)	61 (7.43%)		
Family history of chronic diseases [n (%)]	793 (26.59%)	502 (23.23%)	291 (35.44%)	44.849	<0.001
BMI (kg/m^2^) (M,P25,P75)	23.09 [20.95;25.38]	22.68 [20.71;24.86]	24.46 [22.00;26.90]	−11.445	<0.001
BMI Level [n (%)]					<0.001
Underweight (<18.5 kg/m^2^)	200 (6.71%)	171 (7.91%)	29 (3.53%)	138.757	
Normal (18.5–23.9 kg/m^2^)	1588 (53.25%)	1252 (57.94%)	336 (40.93%)		
Overweight (24.0–27.9 kg/m^2^)	957 (32.09%)	620 (28.69%)	337 (41.05%)		
Obese (≥28.0 kg/m^2^)	237 (7.95%)	118 (5.46%)	119 (14.49%)		
Adequate physical activity [n (%)]	2103 (70.52%)	1565 (72.42%)	538 (65.53%)	13.259	<0.001
Smoking [n (%)]	790 (26.49%)	591 (27.35%)	199 (24.24%)	2.797	0.094
Drinking [n (%)]	1008 (33.80%)	723 (33.46%)	285 (34.71%)	0.366	0.545
Multimorbidity [n (%)]	557 (18.68%)	80 (3.70%)	477 (58.10%)	1155.473	<0.001
Polypharmacy [n (%)]	88 (2.95%)	5 (0.23%)	83 (10.11%)	199.280	<0.001
HBS [$\bar{X}$ (SD)]	22.58 (5.54)	22.62 (5.58)	22.47 (5.43)	0.660	0.509
LBS [$\bar{X}$ (SD)]	40.48 (8.72)	40.49 (8.69)	40.44 (8.81)	0.144	0.886
DQD [$\bar{X}$ (SD)]	63.06 (9.38)	63.11 (9.38)	62.91 (9.39)	0.519	0.604

## Data Availability

The data are not permitted to be disclosed according to the National Institute for Nutrition and Health, Chinese Center for Disease Control and Prevention.

## Supplementary Materials

The following supporting information can be downloaded at: https://www.mdpi.com/article/10.3390/nu18010043/s1, Supplementary Text: Detailed Descriptions of Figures; Table S1: Components of the DBI-2022 Dietary Variety Score; Table S2: Scoring Criteria of the DBI-2022; Table S3: Distribution of Participants across Dietary Quality Grades for Each DBI-2022 Dimension, Stratified by Subgroup Characteristics n (%); Table S4: Distribution of Participants across Score Quartiles for Each DBI-2022 Dimension, Stratified by Subgroup Characteristics n (%); Table S5: DBI-2022 Scores by Participant Characteristics $\bar{X}$ (SD); Table S6: Mean Intake and Score of Dietary Components by Participant Characteristics $\bar{X}$ (SD); Table S7: Correlations between dietary components and diet quality indices; Table S8: Subgroup Analysis of the Association Between DBI-2022 and Hypertension; Table S9: Association Between DBI-2022 and Hypertension Risk: Main and Sensitivity Analyses.
