# Supplementary material for: Application of the China Diet Balance Index (DBI-2022) in a Region with a High-Quality Dietary Pattern and Its Association with Hypertension: A Cross-Sectional Study in the Lingnan Population
Source: Nutrients. 2025 Dec 22;18(1):43. doi: 10.3390/nu18010043 (PMC12787480; doi:10.3390/nu18010043)
Supplement: Supplementary file 1 [file nutrients-18-00043-s001.zip › nutrients-4021214-supplementary.pdf]

Supplementary File

Application of the China Diet Balance Index (DBI-2022) in a Region with a High-Quality Dietary Pattern and its Association with Hypertension: A Cross-Sectional Study in the Lingnan Population

Weihua Dong 1,†, Jian Wen 2,†, Xiaona Zhang 1, Weiyi Gong 1, Ping Gan 2, Panpan Huang 2, Jiaqi Li 2, Rongzhen Li 2, Pengkun Song 1,3, and Gangqiang Ding 2,3,**

Supplementary Text: Detailed Descriptions of Figures

Figure 2. Distribution of dietary quality scores.
(a) Stacked percentage bar charts illustrating the distribution of participants across dietary quality categories (Acceptable, Mild, Moderate, Severe) for each DBI-2022 dimension (LBS, HBS, and DQD from top to bottom); (b) Stacked percentage bar charts showing the distribution of participants across quartiles (Q1 – Q4) of the scores for each DBI-2022 dimension (LBS, HBS, and DQD from top to bottom).

Figure 3. Mean scores and component intake across subgroups.
(a) A three-layer circular stacked bar chart displaying the DBI-2022 mean scores for each dimension (DQD, HBS, and LBS from outermost to innermost layer) across different population subgroups. The central bar chart represents the mean scores for the entire population; (b) A circular bar chart illustrating the intake and scores for each DBI-2022 dietary component across different population subgroups. Notably, the Dietary Variety Score is presented on a reverse scale (negative values) to differentiate it from intake-based metrics.

Figure 4. Contribution weights and intercorrelations of dietary components.
(a) Heatmap illustrating the contribution weights of each DBI-2022 dietary component to the DQD score for the total population and subgroups stratified by hypertension status. Red cells indicate a positive weight, while blue cells indicate a negative weight; (b) The triangular heatmap shows the intercorrelations between the DBI-2022 dietary components. The curves overlaid on the heatmap represent the correlations between each component and the three DBI-2022 dimensions (DQD, HBS, LBS). The color of the curve indicates the direction of the correlation (red for positive, blue for negative), the line type denotes statistical significance (solid for significant, dashed for non-significant), and the thickness reflects the strength of the correlation.

Contribution Coefficient: Represents the magnitude of the weight contributed by each dietary component to the DQD score.
Correlation Coefficient 1: Represents the strength of the intercorrelations among the dietary components.
Correlation Coefficient 2: Represents the strength of the correlation between each dietary component and the different dimensions of the DBI-2022.

Figure 6. Subgroup analysis and interaction effects.
In the "Interaction Effect / P for Interaction" column, the values for each subgroup represent the interaction effect coefficient (top) and the P-value for the interaction (bottom).

Table S1 Components of the DBI-2022 Dietary Variety Score

Food Subgroups	Score	Representative Foods
F1 - Rice and products	-1, 0	Steamed/boiled rice, rice products
F2 - Wheat and products	-1, 0	Wheat bun, wheat noodles, wheat pancake
F3 - Corn, coarse grains and products, starchy roots and products	-1, 0	Corn, barley grain, foxtail millet, buckwheat, sweet potato, yam, taro, potato, green bean, red bean
F4 - Dark-colored vegetables	-1, 0	Spinach, carrot, tomato
F5 - Light-colored vegetables	-1, 0	Cabbage, cucumber, pickles
F6 - Fruit	-1, 0	Fresh and dried fruit
F7 - Soybean and products	-1, 0	Soybean, black bean, bean curd
F8 - Milk and dairy products	-1, 0	Milk, milk powder, cheese
F9 - Red meat and products	-1, 0	Beef, pork, lamb, liver, sausage
F10 - Poultry and game	-1, 0	Chicken, duck, rabbit
F11 - Egg	-1, 0	Hen egg, duck egg
F12 - Fish and shellfish	-1, 0	Fish, shrimp, mussel

Table S2 Scoring Criteria of the DBI-2022

		Intake Range by Energy Intake Level										
Subgroup	Score	4180kJ/1000 kcal	5020kJ/1200 kcal	5860kJ/1400 kcal	6700kJ/1600 kcal	7550kJ/1800 kcal	8350kJ/2000 kcal	9200kJ/2200 kcal	10050kJ/2400 kcal	10900kJ/2600 kcal	11700kJ/2800 kcal	12550kJ/3000 kcal
Cereal	(-12) - 12	0g = -12; 75-95g = 0; >170g = 12	<15g = -12; 90-110g = 0; >185g = 12	0g = -12; 125-175g = 0; >300g = 12	<10g = -12; 175-225g = 0; >390g = 12	<35g = -12; 200-250g = 0; >415g = 12	<5g = -12; 225-275g = 0; >495g = 12	<30g = -12; 250-300g = 0; >520g = 12	0g = -12; 275-325g = 0; >600g = 12	<50g = -12; 325-375g = 0; >650g = 12	<75g = -12; 350-400g = 0; >675g = 12	<100g = -12; 375-425g = 0; >700g = 12
Vegetable	(-6) - 0	≥200g = 0; 160-199g = -1 (score decreased by 1 with 40g intake reduction); 0g = -6	≥250g = 0; 200-249g = -1 (score decreased by 1 with 50g intake reduction); 0g = -6	≥300g = 0; 240-299g = -1 (score decreased by 1 with 60g intake reduction); 0g = -6		≥400g = 0; 320-399g = -1 (score decreased by 1 with 80g intake reduction); 0g = -6	≥450g = 0; 360-449g = -1 (score decreased by 1 with 90g intake reduction); 0g = -6		≥500g = 0; 400-499g = -1 (score decreased by 1 with 100g intake reduction); 0g = -6			≥600g = 0; 480-599g = -1 (score decreased by 1 with 120g intake reduction); 0g = -6
Fruit	(-6) - 0	0g = -6; ≥150g = 0; Score decreased by 1 for every 30g decrease in intake between 120-149g			0g = -6; ≥200g = 0; Score decreased by 1 for every 40g decrease in intake between 160-199g		0g = -6; ≥300g = 0; Score decreased by 1 for every 60g decrease in intake between 240-299g		0g = -6; ≥350g = 0; Score decreased by 1 for every 70g decrease in intake between 280-349g		0g = -6; ≥400g = 0; Score decreased by 1 for every 80g decrease in intake between 320-399g	
Dairy	(-6) - 0	≥500g = 0; Score decreased by 1 for every 100g decrease in intake; 0g = -6		≥350g = 0; Score decreased by 1 for every 70g decrease in intake; 0g = -6	≥300g = 0; Score decreased by 1 for every 60g decrease in intake; 0g = -6							

Soybean and products	(-6) - 0	0g = -6; ≥5g = 0; Score decreased by 1 for every 1g decrease in intake	0g = -6; ≥15g = 0; Score decreased by 1 for every 3g decrease in intake					0g = -6; ≥25g = 0; Score decreased by 1 for every 5g decrease in intake			
Red meat and products, poultry and game	(-4) - 4	0g = -3 ; 1-5g = -2; 6-10g = -1; 11-20g = 0; 21-25g = 1; 26-30g = 2; 31-35g = 3; >35g = 4	0g = -4 ; 1-5g = -3; 6-10g = -2; 11-15g = -1; 16-35g = 0; 36-40g = 1; 41-45g = 2; 46-50g = 3; >50g = 4	0g = -4 ; 1-10g = -3; 11-20g = -2; 21-30g = -1; 31-50g = 0; 51-60g = 1; 61-70g = 2; 71-80g = 3; >80g = 4		0g = -4 ; 1-15g = -3; 16-30g = -2; 31-45g = -1; 46-55g = 0; 56-70g = 1; 71-85g = 2; 85-100g = 3; >100g = 4		0g = -4 ; 1-20g = -3; 21-40g = -2; 41-60g = -1; 61-90g = 0; 91-110g = 1; 111-130g = 2; 131-150g = 3; >150g = 4		0g = -4 ; 1-25g = -3; 26-50g = -2; 51-75g = -1; 76-125g = 0; 126-150g = 1; 151-175g = 2; 176-200g = 3; >200g = 4	
Fish and shrimp	(-4) - 0	0g = -4; 1-4g = -3; 5-9g = -2; 10-14g = -1; ≥15g = 0	<5g = -4; 5-9g = -3; 10-14g = -2; 15-19g = -1; ≥20g = 0	<10g = -4; 10-19g = -3; 20-29g = -2; 30-39g = -1; ≥40g = 0		<5g = -4; 5-19g = -3; 20-34g = -2; 35-49g = -1; ≥50g = 0		0g = -4; 1-24g = -3; 25-49g = -2; 50-74g = -1; ≥75g = 0		<25g = -4; 25-49g = -3; 50-74g = -2; 75-99g = -1; ≥100g = 0	<50g = -4; 50-74g = -3; 75-99g = -2; 100-124g = -1; ≥125g = 0
Egg	(-4) - 4	0g = -4; 1-5g = -3 ;6-10g = -2;11-15g = -1; 16-25g = 0; 26-30g = 1; 31-35g = 2; 36-40g = 3; >40g = 4	<5g = -4; 6-10g = -3; 11-15g= -2;16-20g = -1; 21-30g = 0; 31-35g = 1; 36-40g = 2; 41-45g = 3; >45g = 4		0g = -4; 1-10g= -3;11-15g = -2; 21-30g = -1; 31-50g = 0; 51-60g = 1; 61-70g = 2; 71-80g = 3; >80g = 4		0g = -4; 56-70g = 1	1-15g = -3; 71-85g = 2;	16-30g = -2;85-100g = 3	31-45g = -1;>100g = 4	46-55g = 0
Alcoholic beverages	0 - 6	Male：≤25g=0, 26-40g=1, 6-100g score increased 1 with intake amount increased 15g, >100g=6 (25g alcohol=750ml beer or 250ml wine or 75g liquor (38°) or 50g liquor (> 38°)) Female: ≤15g=0, 16g=1, 16-25g=1, score increased 1 with intake amount increased 10g, >65g=6 (15g alcohol=450ml beer or 150ml wine or 50g liquor (38°) or 30g liquor (> 38°))									
Cooking oil	0 - 6	≤20g = 0; 21-25g = 1; >45g = 6	≤25g = 0; 26-30g = 1; >50g = 6					≤30g = 0; 31-35g = 1; >55g = 6		≤35g = 0; 36-40g = 1; >60g = 6	
Added sugar	0 - 6	≤25g=0 ; 26g=1 score increased 1 with intake amount increased 5g ; >50g=6									
Salt	0 - 6	<2g = 0 ; 2-3g=1 score increased 1 with intake amount	<3g= 0 ; 3-4g=1 score increased 1 with intake	<4g = 0 ; 4-5g=1 score increased 1 with	<5g = 0; 5-6g = 1 ,Score increased by 1 for every 2g increase in intake; >15g = 6						

		increased 2g ; >12g=6	amount increased 2g ; >13g = 6	intake amount increased 2g ; >14g = 6	
Diet variety	(-12) - 0	≥12 kinds of food (soybean ≥5g, others ≥25g) = 0; score decreased 1 with decreased 1 kinds of food ; 0 kinds of food = -12			
Drinking water	(-12) - 0	≥1200ml = 0; Score decreased by 1 for every 100ml decrease in intake; <100ml = -12			

Table S3 Distribution of Participants across Dietary Quality Grades for Each DBI-2022 Dimension, Stratified by Subgroup Characteristics n（%）

		DQD			HBS				LBS	
	Mild	Moderate	Severe	Acceptable	Mild	Moderate	Severe	Mild	Moderate	Severe
Total	21（0.71%）	77（25.92%）	2188（73.37%）	62(2.08%)	568(19.05%)	1628(54.59%)	724(24.28%)	337(11.3%)	1479(49.60%)	1166(39.10%)
Gender										
Female	13(61.90%)	421(54.46%)	1135(51.87%)	25(40.32%)	318(55.99%)	877(53.87%)	349(48.20%)	183(54.30%)	746(50.44%)	640(54.89%)
Male	8(38.10%)	352(45.54%)	1053(48.13%)	37(59.68%)	250(44.01%)	751(46.13%)	375(51.80%)	154(45.70%)	733(49.56%)	526(45.11%)
Statistic/P Overall/Trend		2.268/0.322/0.148			13.024/0.005/0.067				5.610/0.061/0.243	
Residence										
Urban	20(95.24%)	528(68.31%)	629(28.75%)	31(50.00%)	244(42.96%)	653(40.11%)	249(34.39%)	277(82.20%)	680(45.98%)	220(18.87%)
Rural	1(4.76%)	245(31.69%)	1559(71.25%)	31(50.00%)	324(57.04%)	975(59.89%)	475(65.61%)	60(17.80%)	799(54.02%)	946(81.13%)
Statistic/P Overall/Trend		401.650/<0.001/<0.001			13.862/0.003/<0.001				490.858/<0.001/<0.001	
Smoke										
Never smoker	19(90.48%)	581(75.16%)	1592(72.76%)	42(67.74%)	432(76.06%)	1220(74.94%)	498(68.78%)	264(78.34%)	1049(70.93%)	879(75.39%)
Current smoker	2(9.52%)	192(24.84%)	596(27.24%)	20(32.26%)	136(23.94%)	408(25.06%)	226(31.22%)	73(21.66%)	430(29.07%)	287(24.61%)
Statistic/P Overall/Trend		4.818/0.090/0.073			12.958/0.005/0.017				11.211/0.004/0.721	
Drink										
Never drinker	14(66.67%)	473(61.19%)	1487(67.96%)	39(62.90%)	401(70.60%)	1108(68.06%)	426(58.84%)	175(51.93%)	973(65.79%)	826(70.84%)
Current drinker	7(33.33%)	300(38.81%)	701(32.04%)	23(37.10%)	167(29.40%)	520(31.94%)	298(41.16%)	162(48.07%)	506(34.21%)	340(29.16%)
Statistic/P Overall/Trend		11.707/0.003/0.001			25.254/<0.001/<0.001				42.007/<0.001/<0.001	

Physical activity										
Inadequate	4(19.05%)	232(30.01%)	643(29.39%)	19(30.65%)	179(31.51%)	493(30.28%)	188(25.97%)	93(27.60%)	420(28.40%)	366(31.39%)
Adequate	17(80.95%)	541(69.99%)	1545(70.61%)	43(69.35%)	389(68.49%)	1135(69.72%)	536(74.03%)	244(72.40%)	1059(71.60%)	800(68.61%)
Statistic/P Overall/Trend		1.214/0.545/0.984				5.974/0.113/0.032			3.454/0.178/0.078	
BMI Level										
Underweight	1(4.76%)	35(4.53%)	164(7.50%)	4(6.45%)	39(6.87%)	106(6.51%)	51(7.04%)	13(3.86%)	86(5.81%)	101(8.66%)
Normal	9(42.86%)	380(49.16%)	1199(54.80%)	31(50.00%)	306(53.87%)	890(54.67%)	361(49.86%)	158(46.88%)	753(50.91%)	677(58.06%)
Overweight	10(47.62%)	276(35.71%)	671(30.67%)	20(32.26%)	176(30.99%)	518(31.82%)	243(33.56%)	126(37.39%)	505(34.14%)	326(27.96%)
Obese	1(4.76%)	82(10.61%)	154(7.04%)	7(11.29%)	47(8.27%)	114(7.00%)	69(9.53%)	40(11.87%)	135(9.13%)	62(5.32%)
Statistic/P Overall/Trend		26.994/<0.001/<0.001				8.387/0.496/0.369			51.990/<0.001/<0.001	
Hypertension										
No	16(76.19%)	562(72.70%)	1583(72.35%)	46(74.19%)	403(70.95%)	1171(71.93%)	541(74.72%)	241(71.51%)	1084(73.29%)	836(71.70%)
Yes	5(23.81%)	211(27.30%)	605(27.65%)	16(25.81%)	165(29.05%)	457(28.07%)	183(25.28%)	96(28.49%)	395(26.71%)	330(28.30%)
Statistic/P Overall/Trend		0.183/0.913/0.764				2.832/0.418/0.193			1.005/0.605/0.718	

***P-Overall: P-value for between-group differences; P-Trend: P-value for trend test**

Table S4 Distribution of Participants across Score Quartiles for Each DBI-2022 Dimension, Stratified by Subgroup Characteristics n（%）

	DQD				HBS				LBS			
	Q1	Q2	Q3	Q4	Q1	Q2	Q3	Q4	Q1	Q2	Q3	Q4
Total	731（24.51%）	728（24.41%）	733（24.58%）	790（26.49）	664（22.27%）	707（23.71%）	807（27.06%）	804（26.96%）	665(22.30%)	759(25.45%)	756(25.35%)	802(26.89%)
Gender												
Female	395(54.04%)	370(50.82%)	376(51.30%)	428(54.18%)	364(54.82%)	386(54.60%)	427(52.91%)	392(48.76%)	347(52.18%)	382(50.33%)	397(52.51%)	443(55.24%)
Male	336(45.96%)	358(49.18%)	357(48.70%)	362(45.82%)	300(45.18%)	321(45.40%)	380(47.09%)	412(51.24%)	318(47.82%)	377(49.67%)	359(47.49%)	359(44.76%)
Statistic/P Overall/Trend	2.813/0.421/0.880				7.238/0.065/0.014				3.855/0.278/0.144			
Residence												
Urban	514(70.31%)	331(45.47%)	198(27.01%)	134(16.96%)	290(43.67%)	281(39.75%)	315(39.03%)	291(36.19%)	490(73.68%)	336(44.27%)	216(28.57%)	135(16.83%)
Rural	217(29.69%)	397(54.53%)	535(72.99%)	656(83.04%)	374(56.33%)	426(60.25%)	492(60.97%)	513(63.81%)	175(26.32%)	423(55.73%)	540(71.43%)	667(83.17%)
Statistic/P Overall/Trend	517.189/<0.001/<0.001				8.612/0.035/0.004				542.755/<0.001/<0.001			
Smoke												
Never smoker	554(75.79%)	521(71.57%)	533(72.71%)	584(73.92%)	503(75.75%)	537(75.95%)	592(73.36%)	560(69.65%)	496(74.59%)	534(70.36%)	549(72.62%)	613(76.43%)
Current smoker	177(24.21%)	207(28.43%)	200(27.29%)	206(26.08%)	161(24.25%)	170(24.05%)	215(26.64%)	244(30.35%)	169(25.41%)	225(29.64%)	207(27.38%)	189(23.57%)
Statistic/P Overall/Trend	3.666/0.300/0.558				10.041/0.018/0.003				8.103/0.044/0.216			
Drink												
Never drinker	448(61.29%)	493(67.72%)	497(67.80%)	536(67.85%)	461(69.43%)	507(71.71%)	527(65.30%)	479(59.58%)	391(58.80%)	502(66.14%)	502(66.40%)	579(72.19%)
Current drinker	283(38.71%)	235(32.28%)	236(32.20%)	254(32.15%)	203(30.57%)	200(28.29%)	280(34.70%)	325(40.42%)	274(41.20%)	257(33.86%)	254(33.60%)	223(27.81%)
Statistic/P Overall/Trend	10.442/0.015/0.011				28.739/<0.001/<0.001				29.181/<0.001/<0.001			
Physical activity												
Inadequate	219(29.96%)	212(29.12%)	196(26.74%)	252(31.90%)	213(32.08%)	222(31.40%)	230(28.50%)	214(26.62%)	187(28.12%)	215(28.33%)	216(28.57%)	261(32.54%)
Adequate	512(70.04%)	516(70.88%)	537(73.26%)	538(68.10%)	451(67.92%)	485(68.60%)	577(71.50%)	590(73.38%)	478(71.88%)	544(71.67%)	540(71.43%)	541(67.46%)

Statistic/P Overall/Trend	4.997/0.172/0.601				6.953/0.073/0.010				4.998/0.172/0.063			
BMI Level												
Underweight	30(4.10%)	45(6.18%)	58(7.91%)	67(8.48%)	43(6.48%)	49(6.93%)	53(6.57%)	55(6.84%)	29(4.36%)	42(5.53%)	57(7.54%)	72(8.98%)
Normal	359(49.11%)	358(49.18%)	426(58.12%)	445(56.33%)	358(53.92%)	390(55.16%)	442(54.77%)	398(49.50%)	307(46.17%)	391(51.52%)	425(56.22%)	465(57.98%)
Overweight	265(36.25%)	250(34.34%)	207(28.24%)	235(29.75%)	205(30.87%)	219(30.98%)	257(31.85%)	276(34.33%)	252(37.89%)	252(33.20%)	225(29.76%)	228(28.43%)
Obese	77(10.53%)	75(10.30%)	42(5.73%)	43(5.44%)	58(8.73%)	49(6.93%)	55(6.82%)	75(9.33%)	77(11.58%)	74(9.75%)	49(6.48%)	37(4.61%)
Statistic/P Overall/Trend	54.100/<0.001/<0.001				9.680/0.377/0.213				64.575/<0.001/<0.001			
Hypertension												
No	528(72.23%)	526(72.25%)	526(71.76%)	581(73.54%)	475(71.54%)	516(72.98%)	574(71.13%)	596(74.13%)	478(71.88%)	555(73.12%)	559(73.94%)	569(70.95%)
Yes	203(27.77%)	202(27.75%)	207(28.24%)	209(26.46%)	189(28.46%)	191(27.02%)	233(28.87%)	208(25.87%)	187(28.12%)	204(26.88%)	197(26.06%)	233(29.05%)
Statistic/P Overall/Trend	0.681/0.878/0.622				2.222/0.528/0.414				2.031/0.566/0.730			

***P-Overall: P-value for between-group differences; P-Trend: P-value for trend test**

Table S5 DBI-2022 Scores by Participant Characteristics $\overline{X}(SD)$

	DQD	HBS	LBS
Total	63.06 (9.38)	22.58 (5.54)	40.48 (8.72)
Gender			
Female	62.99 (9.54)	22.34 (5.38)	40.65 (8.89)
Male	63.13 (9.20)	22.85 (5.70)	40.28 (8.54)
Statistic/P Value	0.395/0.693	2.505/<0.05	-1.171/0.242
Residence			
Urban	58.01 (9.21)	22.25 (5.79)	35.76 (8.64)
Rural	66.35 (7.92)	22.80 (5.36)	43.55 (7.29)
Statistic/P Value	-25.530/<0.001	-2.623/<0.01	-25.564/<0.001
Smoke			
Never smoker	62.95 (9.57)	22.41 (5.49)	40.54 (8.95)
Current smoker	63.35 (8.85)	23.05 (5.64)	40.30 (8.07)
Statistic/P Value	-1.051/0.293	-2.747/<0.01	0.706/0.480
Drink			
Never drinker	63.41 (9.19)	22.21 (5.40)	41.20 (8.51)
Current drinker	62.37 (9.72)	23.31 (5.73)	39.06 (8.97)
Statistic/P Value	2.810/<0.05	-5.033/<0.01	6.251/<0.001
Physical Activity			
Inadequate	63.23 (9.49)	22.26 (5.44)	40.97 (9.00)
Adequate	62.98 (9.34)	22.71 (5.57)	40.27 (8.60)
Statistic/P Value	0.646/0.518	-2.043/<0.05	1.949/0.051
Hypertension			
No	63.11 (9.38)	22.62 (5.58)	40.49 (8.69)
Yes	62.91 (9.39)	22.47 (5.43)	40.44 (8.81)
Statistic/P Value	0.519/0.604	0.660/0.509	0.144/0.886
BMI Level			
Underweight	65.34 (9.42)	22.60 (5.28)	42.73 (8.61)
Normal	63.67 (9.16)	22.44 (5.45)	41.23 (8.61)
Overweight	62.26 (9.51)	22.81 (5.59)	39.46 (8.70)
Obese	60.25 (9.47)	22.59 (6.06)	37.66 (8.54)
Statistic/P Value	48.129/<0.001	3.633/0.304	66.040/<0.001

Table S6 Mean Intake and Score of Dietary Components by Participant Characteristics $\overline{X}(SD)$

	Cereal（g）	Vegetable（g）	Fruit（g）	Dairy（g）	Animal Food（g）	Aquatic Products（g）	Egg（g）	Soybean（g）	Oil（g）	Alcohol（g）	Sugar（g）	Drinking Water（ml）	Salt（g）	Diet Variety
Overall	260.89 (127.39)	306.96 (160.77)	34.36 (69.69)	11.28 (49.31)	147.13 (91.33)	41.87 (53.11)	15.11 (23.74)	9.95 (20.33)	40.50 (30.75)	6.53 (19.97)	1.26 (4.28)	981.29 (666.87)	19.46 (16.40)	-5.86 (1.79)
Female	259.66 (127.80)	318.34 (157.89)	39.67 (73.93)	13.06 (53.86)	141.34 (88.01)	41.35 (52.84)	15.73 (24.32)	10.28 (20.96)	39.81 (30.49)	1.07 (6.51)	1.23 (4.14)	878.65 (580.56)	19.12 (16.19)	-5.81 (1.80)
Male	262.25 (126.97)	294.31 (163.04)	28.46 (64.17)	9.29 (43.65)	153.56 (94.50)	42.44 (53.42)	14.43 (23.07)	9.58 (19.61)	41.25 (31.03)	12.59 (26.92)	1.29 (4.45)	1095.26 (734.78)	19.83 (16.63)	-5.92 (1.78)
Statistic/P Value	0.556/0.578	-4.079/<0.001	-4.432/<0.001	-2.112/0.034	3.644/<0.001	0.563/0.574	-1.493/0.136	-0.948/0.343	1.274/0.203	15.683/<0.001	0.366/0.714	8.866/<0.001	1.173/0.241	-1.645/0.100
Urban	238.17 (115.65)	336.69 (173.77)	61.64 (92.81)	26.40 (74.36)	149.51 (88.97)	47.72 (53.34)	21.99 (26.85)	12.27 (20.89)	36.04 (27.63)	4.32 (15.61)	1.50 (4.45)	1125.57 (687.23)	19.25 (16.32)	-4.95 (1.85)
Rural	275.70 (132.45)	287.57 (148.58)	16.58 (40.10)	1.41 (12.95)	145.58 (92.83)	38.05 (52.62)	10.63 (20.26)	8.43 (19.81)	43.40 (32.30)	7.97 (22.25)	1.10 (4.17)	887.20 (636.05)	19.59 (16.46)	-6.46 (1.48)
Statistic/P Value	-8.173/<0.001	7.981/<0.001	15.726/<0.001	11.420/<0.001	1.158/0.247	4.864/<0.001	12.395/<0.001	5.015/<0.001	-6.647/<0.001	-5.267/<0.001	2.424/0.015	9.532/<0.001	-0.553/0.580	23.503/<0.001
Current smoker	257.43 (125.90)	281.59 (154.98)	20.73 (48.21)	5.79 (32.37)	156.82 (97.80)	44.16 (54.32)	13.51 (22.45)	8.83 (18.31)	41.80 (30.49)	16.25 (29.38)	1.20 (4.22)	1147.59 (779.74)	20.23 (16.33)	-5.99 (1.69)
Never smoker	262.13 (127.93)	316.10 (161.87)	39.27 (75.36)	13.25 (54.01)	143.64 (88.65)	41.04 (52.65)	15.69 (24.16)	10.35 (21.00)	40.02 (30.84)	3.03 (13.61)	1.28 (4.31)	921.35 (610.38)	19.18 (16.42)	-5.82 (1.82)
Statistic/P Value	0.896/0.371	5.301/<0.001	7.881/<0.001	4.580/<0.001	-3.327/<0.001	-1.397/0.163	2.288/0.022	1.928/0.054	-1.400/0.162	-12.189/<0.001	0.469/0.639	-7.381/<0.001	-1.555/0.120	2.426/0.015
Current drinker	258.04 (128.33)	315.26 (171.70)	40.49 (79.24)	13.40 (52.61)	154.66 (96.12)	45.93 (56.32)	16.61 (24.94)	10.33 (20.76)	42.24 (30.80)	18.97 (30.40)	1.29 (4.30)	1111.51 (697.87)	19.69 (16.36)	-5.67 (1.85)
Never drinker	262.34 (126.92)	302.72 (154.77)	31.23 (64.07)	10.19 (47.52)	143.28 (88.56)	39.79 (51.28)	14.35 (23.07)	9.75 (20.11)	39.61 (30.69)	0.18 (3.40)	1.24 (4.28)	914.79 (640.53)	19.34 (16.43)	-5.96 (1.75)
Statistic/P Value	0.869/0.385	-1.949/0.051	-3.211/0.001	-1.625/0.104	-3.137/0.002	-2.904/0.004	-2.400/0.017	-0.730/0.465	-2.208/0.027	-19.560/<0.001	-0.271/0.787	-7.483/<0.001	-0.561/0.575	-4.170/<0.001
Adequate physical activity	257.42 (128.92)	298.94 (167.23)	34.83 (72.48)	14.38 (56.69)	143.90 (90.31)	37.53 (50.62)	15.83 (24.76)	9.83 (20.37)	37.79 (29.50)	6.30 (20.56)	1.10 (3.71)	948.81 (639.53)	18.92 (15.96)	-5.95 (1.83)

Inadequate physical activity	262.34 (126.76)	310.31 (157.92)	34.17 (68.51)	9.98 (45.83)	148.48 (91.74)	43.68 (54.02)	14.81 (23.30)	10.22 (20.23)	41.62 (31.20)	6.63 (19.72)	1.32 (4.50)	994.86 (677.67)	19.68 (16.58)	-5.83 (1.77)
Statistic/P Value	-0.954/0.340	-1.720/0.086	0.231/0.817	2.041/0.041	-1.257/0.209	-2.962/0.003	1.040/0.298	0.478/0.633	-3.177/0.002	-0.408/0.683	-1.437/0.151	-1.762/0.078	-1.168/0.243	-1.709/0.088
Hypertension	263.23 (133.36)	314.95 (170.38)	35.55 (70.87)	13.23 (53.84)	140.09 (84.42)	40.67 (52.16)	15.48 (24.77)	10.1 (20.19)	38.74 (30.24)	8.34 (22.89)	1.05 (3.49)	977.10 (632.04)	19.76 (17.31)	-5.81 (1.85)
Non-Hypertension	259.99 (125.07)	303.92 (156.90)	33.91 (69.25)	10.53 (47.47)	149.80 (93.70)	42.32 (53.47)	14.97 (23.34)	9.89 (20.39)	41.16 (30.92)	5.84 (18.70)	1.34 (4.55)	982.88 (679.78)	19.34 (16.05)	-5.88 (1.77)
Statistic/P Value	-0.600/0.548	-1.613/0.107	-0.568/0.570	-1.262/0.207	2.719/0.007	0.769/0.442	-0.515/0.607	-0.254/0.799	1.938/0.053	-2.790/0.005	1.842/0.066	0.218/0.827	-0.610/0.542	-0.891/0.373
Underweight	258.25 (123.85)	292.61 (157.52)	23.69 (55.90)	10.73 (46.45)	143.66 (85.93)	48.32 (58.54)	10.90 (20.15)	6.51 (15.00)	37.60 (27.43)	5.80 (18.57)	1.74 (5.33)	885.00 (708.72)	19.28 (14.23)	-6.15 (1.64)
Normal	264.10 (125.27)	299.70 (158.30)	32.00 (68.42)	9.65 (45.78)	147.33 (93.14)	40.84 (51.95)	13.79 (22.50)	9.29 (19.09)	39.81 (30.19)	7.41 (21.61)	1.17 (4.21)	945.47 (673.54)	18.63 (15.70)	-5.99 (1.74)
Overweight	258.17 (128.23)	321.66 (166.99)	37.35 (71.30)	12.25 (52.13)	148.82 (90.37)	41.06 (52.55)	17.79 (26.24)	10.92 (22.38)	41.40 (31.96)	5.78 (18.33)	1.34 (4.34)	1026.54 (631.27)	20.12 (17.04)	-5.69 (1.86)
Obese	252.56 (140.57)	308.32 (150.67)	47.13 (79.42)	18.68 (60.98)	141.89 (87.55)	46.58 (57.75)	16.71 (22.91)	13.34 (22.85)	43.83 (31.92)	4.25 (15.24)	1.13 (3.49)	1119.83 (696.35)	22.46 (19.46)	-5.43 (1.84)
Statistic/P Value	6.656/0.084	14.394/0.002	18.556<0.001	10.716/0.013	1.902/0.593	4.456/0.216	30.678/<0.001	16.600/<0.001	6.484/0.090	3.677/0.299	4.792/0.188	40.812/<0.001	13.891/0.003	37.642/<0.001

Table S7 Correlations between dietary components and diet quality indices

Correlated Variables	Correlation Coefficient (r)	95% CI Lower	95% CI Upper	*P* Value
Cereal—Vegetable	0.175	0.14	0.209	< 0.001
Cereal—Fruit	0.028	-0.008	0.064	0.122
Cereal—Dairy	-0.075	-0.11	-0.039	< 0.001
Cereal—Soybean	-0.003	-0.038	0.033	0.889
Cereal—Animal Food	0.131	0.096	0.166	< 0.001
Cereal—Aquatic Product	0.025	-0.011	0.061	0.167
Cereal—Egg	-0.02	-0.056	0.016	0.282
Cereal—Oil	0.114	0.079	0.149	< 0.001
Cereal—Alcohol	-0.004	-0.04	0.031	0.808
Cereal—Sugar	-0.021	-0.056	0.015	0.263
Cereal—Salt	0.047	0.012	0.083	0.010
Cereal—Diet Variety	0.029	-0.007	0.065	0.116
Cereal—Drinking Water	-0.091	-0.127	-0.056	< 0.001
Vegetable—Fruit	0.231	0.197	0.265	< 0.001
Vegetable—Dairy	0.096	0.06	0.132	< 0.001
Vegetable—Soybean	0.095	0.059	0.13	< 0.001
Vegetable—Animal Food	0.277	0.244	0.31	< 0.001
Vegetable—Aquatic Product	0.027	-0.009	0.063	0.135
Vegetable—Egg	0.132	0.097	0.167	< 0.001
Vegetable—Oil	0.08	0.044	0.116	< 0.001
Vegetable—Alcohol	-0.001	-0.036	0.035	0.976
Vegetable—Sugar	0.087	0.051	0.122	< 0.001
Vegetable—Salt	0.062	0.026	0.098	< 0.001
Vegetable—Diet Variety	0.296	0.263	0.328	< 0.001

Vegetable—Drinking Water	0.085	0.049	0.121	< 0.001
Fruit—Dairy	0.238	0.203	0.271	< 0.001
Fruit—Soybean	0.154	0.119	0.189	< 0.001
Fruit—Animal Food	0.055	0.02	0.091	0.002
Fruit—Aquatic Product	0.095	0.059	0.13	< 0.001
Fruit—Egg	0.236	0.202	0.27	< 0.001
Fruit—Oil	-0.04	-0.076	-0.004	0.028
Fruit—Alcohol	0.017	-0.019	0.053	0.362
Fruit—Sugar	0.156	0.121	0.191	< 0.001
Fruit—Salt	0.009	-0.027	0.045	0.613
Fruit—Diet Variety	0.527	0.5	0.552	< 0.001
Fruit—Drinking Water	0.141	0.106	0.176	< 0.001
Dairy—Soybean	0.122	0.087	0.157	< 0.001
Dairy—Animal Food	-0.062	-0.097	-0.026	< 0.001
Dairy—Aquatic Product	0.056	0.021	0.092	0.002
Dairy—Egg	0.183	0.148	0.218	< 0.001
Dairy—Oil	-0.109	-0.145	-0.074	< 0.001
Dairy—Alcohol	0.025	-0.011	0.061	0.173
Dairy—Sugar	0.076	0.041	0.112	< 0.001
Dairy—Salt	-0.033	-0.069	0.003	0.068
Dairy—Diet Variety	0.33	0.298	0.362	< 0.001
Dairy—Drinking Water	0.089	0.054	0.125	< 0.001
Soybean—Animal Food	0.027	-0.009	0.063	0.142
Soybean—Aquatic Product	0.018	-0.018	0.054	0.323
Soybean—Egg	0.122	0.086	0.157	< 0.001
Soybean—Oil	0.052	0.016	0.087	0.005

Soybean—Alcohol	0.01	-0.025	0.046	0.568
Soybean—Sugar	-0.014	-0.049	0.022	0.460
Soybean—Salt	-0.018	-0.054	0.018	0.327
Soybean—Diet Variety	0.423	0.394	0.452	< 0.001
Soybean—Drinking Water	0.093	0.057	0.128	< 0.001
Animal Food—Aquatic Product	-0.04	-0.076	-0.004	0.029
Animal Food—Egg	-0.006	-0.042	0.03	0.759
Animal Food—Oil	0.068	0.032	0.103	< 0.001
Animal Food—Alcohol	0.057	0.021	0.093	0.002
Animal Food—Sugar	0.07	0.034	0.106	< 0.001
Animal Food—Salt	0.142	0.106	0.177	< 0.001
Animal Food—Diet Variety	0.226	0.191	0.259	< 0.001
Animal Food—Drinking Water	0.085	0.049	0.12	< 0.001
Aquatic Product—Egg	0.057	0.021	0.093	0.002
Aquatic Product—Oil	0.044	0.009	0.08	0.015
Aquatic Product—Alcohol	0.036	0	0.072	0.049
Aquatic Product—Sugar	0.065	0.029	0.1	< 0.001
Aquatic Product—Salt	0.07	0.034	0.106	< 0.001
Aquatic Product—Diet Variety	0.346	0.314	0.377	< 0.001
Aquatic Product—Drinking Water	0.143	0.108	0.178	< 0.001
Egg—Oil	0.031	-0.004	0.067	0.086
Egg—Alcohol	0.062	0.026	0.098	< 0.001
Egg—Sugar	0.063	0.027	0.098	< 0.001
Egg—Salt	0.032	-0.004	0.068	0.078
Egg—Diet Variety	0.433	0.404	0.462	< 0.001
Egg—Drinking Water	0.162	0.126	0.196	< 0.001

Oil—Alcohol	0.04	0.004	0.075	0.030
Oil—Sugar	-0.069	-0.104	-0.033	< 0.001
Oil—Salt	0.394	0.364	0.424	< 0.001
Oil—Diet Variety	0.029	-0.007	0.065	0.114
Oil—Drinking Water	0.027	-0.009	0.063	0.138
Alcohol—Sugar	-0.008	-0.044	0.028	0.677
Alcohol—Salt	0.015	-0.021	0.051	0.401
Alcohol—Diet Variety	0.06	0.024	0.095	0.001
Alcohol—Drinking Water	0.147	0.111	0.182	< 0.001
Sugar—Salt	0.054	0.019	0.09	0.003
Sugar—Diet Variety	0.128	0.093	0.163	< 0.001
Sugar—Drinking Water	0.094	0.058	0.13	< 0.001
Salt—Diet Variety	0.075	0.039	0.11	< 0.001
Salt—Drinking Water	0.049	0.013	0.085	0.007
Diet Variety—Drinking Water	0.256	0.222	0.289	< 0.001
DQD—Cereal	0.3	0.266	0.332	< 0.001
DQD—Vegetable	-0.146	-0.181	-0.111	< 0.001
DQD—Fruit	-0.471	-0.499	-0.443	< 0.001
DQD—Dairy	-0.315	-0.347	-0.282	< 0.001
DQD—Soybean	-0.465	-0.493	-0.437	< 0.001
DQD—Animal Food	-0.006	-0.042	0.03	0.734
DQD—Aquatic Product	-0.32	-0.352	-0.287	< 0.001
DQD—Egg	-0.324	-0.356	-0.291	< 0.001
DQD—Oil	0.302	0.269	0.335	< 0.001
DQD—Alcohol	-0.016	-0.051	0.02	0.395
DQD—Sugar	-0.108	-0.143	-0.073	< 0.001

DQD—Salt	0.218	0.184	0.252	< 0.001
DQD—Diet Variety	-0.617	-0.639	-0.594	< 0.001
DQD—Drinking Water	-0.528	-0.553	-0.501	< 0.001
LBS—Cereal	0.026	-0.01	0.062	0.159
LBS—Vegetable	-0.267	-0.3	-0.233	< 0.001
LBS—Fruit	-0.542	-0.567	-0.516	< 0.001
LBS—Dairy	-0.316	-0.348	-0.283	< 0.001
LBS—Soybean	-0.533	-0.558	-0.506	< 0.001
LBS—Animal Food	-0.127	-0.162	-0.091	< 0.001
LBS—Aquatic Product	-0.377	-0.407	-0.346	< 0.001
LBS—Egg	-0.507	-0.533	-0.48	< 0.001
LBS—Oil	-0.049	-0.085	-0.013	0.007
LBS—Alcohol	-0.094	-0.129	-0.058	< 0.001
LBS—Sugar	-0.134	-0.169	-0.099	< 0.001
LBS—Salt	-0.07	-0.106	-0.035	< 0.001
LBS—Diet Variety	-0.802	-0.815	-0.789	< 0.001
LBS—Drinking Water	-0.598	-0.621	-0.574	< 0.001
HBS—Cereal	0.443	0.414	0.472	< 0.001
HBS—Vegetable	0.18	0.145	0.214	< 0.001
HBS—Fruit	0.055	0.019	0.09	0.003
HBS—Dairy	-0.052	-0.088	-0.016	0.005
HBS—Soybean	0.054	0.019	0.09	0.003
HBS—Animal Food	0.19	0.155	0.224	< 0.001
HBS—Aquatic Product	0.06	0.024	0.096	0.001
HBS—Egg	0.262	0.228	0.295	< 0.001
HBS—Oil	0.649	0.628	0.669	< 0.001

HBS—Alcohol	0.125	0.09	0.161	< 0.001
HBS—Sugar	0.018	-0.018	0.054	0.321
HBS—Salt	0.516	0.489	0.542	< 0.001
HBS—Diet Variety	0.204	0.169	0.238	< 0.001
HBS—Drinking Water	0.057	0.021	0.092	0.002

*** All correlations were assessed using the non-parametric Spearman's rank correlation test. Values are presented as correlation coefficients and 95% confidence intervals.**

Table S8 Subgroup Analysis of the Association Between DBI-2022 and Hypertension

Subgroup		Beta	Standard Error	Wald Chi-Square	*P* Value	OR(95%CI)	P for Interaction	Interaction Effect
**Dimension：DQD**								
Gender	Male	0.213	0.094	5.2	0.023	1.238(1.03,1.487)	0.6953	0.959
	Female	0.098	0.09	1.189	0.276	1.103(0.925,1.315)		
Residence	Urban	0.186	0.105	3.162	0.075	1.204(0.981,1.479)	0.2343	0.866
	Rural	0.112	0.083	1.827	0.176	1.118(0.951,1.316)		
BMI Level	Underweight	-0.146	0.342	0.182	0.67	0.865(0.443,1.688)	0.7331	0.975
	Normal	0.112	0.095	1.38	0.24	1.118(0.928,1.347)		
	Overweight	0.331	0.108	9.469	0.002	1.392(1.128,1.719)		
	Obese	-0.099	0.204	0.237	0.626	0.905(0.607,1.351)		
Physical Activity	Inadequate	-0.005	0.118	0.002	0.963	0.995(0.789,1.253)	0.0083	1.36
	adequate	0.242	0.077	9.777	0.002	1.274(1.095,1.483)		
Drink	No	0.088	0.079	1.261	0.261	1.092(0.936,1.274)	0.1037	1.2
	Yes	0.256	0.115	4.972	0.026	1.291(1.031,1.617)		
Smoke	No	0.17	0.076	4.992	0.025	1.186(1.021,1.377)	0.6122	0.94
	Yes	0.107	0.122	0.781	0.377	1.113(0.877,1.413)		
**Dimension：HBS**								
Gender	Male	0.009	0.08	0.011	0.915	1.009(0.862,1.181)	0.8459	0.979
	Female	-0.006	0.084	0.006	0.939	0.994(0.843,1.172)		
Residence	Urban	0.171	0.093	3.417	0.065	1.187(0.99,1.424)	0.0145	0.761
	Rural	-0.137	0.076	3.242	0.072	0.872(0.751,1.012)		
BMI Level	Underweight	-0.3	0.301	0.995	0.319	0.741(0.411,1.336)	0.0451	1.156
	Normal	-0.109	0.084	1.672	0.196	0.897(0.76,1.058)		
	Overweight	0.146	0.099	2.16	0.142	1.157(0.953,1.405)		
	Obese	0.11	0.177	0.385	0.535	1.116(0.789,1.578)		

Physical Activity	Inadequate	-0.236	0.109	4.717	0.03	0.79(0.638,0.977)	0.0705	1.309
	adequate	0.088	0.07	1.584	0.208	1.092(0.952,1.251)		
Drink	No	0.065	0.071	0.839	0.36	1.067(0.928,1.228)	0.1197	0.840
	Yes	-0.131	0.101	1.67	0.196	0.877(0.719,1.07)		
Smoke	No	0.044	0.069	0.405	0.525	1.045(0.913,1.197)	0.2572	0.873
	Yes	-0.104	0.107	0.95	0.33	0.901(0.731,1.111)		
**Dimension：LBS**								
Gender	Male	0.319	0.098	10.587	0.001	1.376(1.135,1.668)	0.2184	0.875
	Female	0.066	0.096	0.471	0.493	1.068(0.885,1.289)		
Residence	Urban	0.119	0.11	1.163	0.281	1.126(0.908,1.397)	0.8206	0.972
	Rural	0.212	0.088	5.767	0.016	1.236(1.04,1.469)		
BMI Level	Underweight	0.523	0.435	1.444	0.229	1.687(0.719,3.96)	0.0571	0.864
	Normal	0.28	0.102	7.585	0.006	1.323(1.084,1.614)		
	Overweight	0.174	0.11	2.497	0.114	1.19(0.959,1.477)		
	Obese	-0.131	0.226	0.336	0.562	0.877(0.564,1.365)		
Physical Activity	Inadequate	0.204	0.133	2.344	0.126	1.226(0.945,1.59)	0.1441	1.187
	adequate	0.208	0.08	6.814	0.009	1.231(1.053,1.439)		
Drink	No	0.091	0.082	1.219	0.27	1.095(0.932,1.286)	0.0297	1.281
	Yes	0.368	0.124	8.873	0.003	1.445(1.134,1.84)		
Smoke	No	0.133	0.08	2.749	0.097	1.142(0.976,1.336)	0.3250	1.130
	Yes	0.313	0.132	5.618	0.018	1.367(1.056,1.771)		

Table S9 Association Between DBI-2022 and Hypertension Risk: Main and Sensitivity Analyses

**Model Scenario**	**Group**	**Hypertension/Non-hypertension,(N)**	**OR(95%CI)**	**Beta**	**Standard Error**	**Wald Chi-Square**	***P* Value**
**Dimension:DQD**							
Original Model	Q1	821/2161					
	Q2		1.4261(0.9763,2.0886)	0.3549	0.1939	3.3519	0.0671
	Q3		2.1169(1.4303,3.1479)	0.7499	0.2011	13.9071	0.0002
	Q4		1.5702(1.052,2.3527)	0.4512	0.2052	4.8358	0.0279
Scenario 1: Exclusion of Prior Diagnosis	Q1	294/2161					
	Q2		1.5617(0.922,2.6687)	0.4458	0.2706	2.7135	0.0995
	Q3		1.8157(1.0528,3.1615)	0.5965	0.2801	4.5359	0.0332
	Q4		1.9579(1.1463,3.3864)	0.6719	0.2759	5.9278	0.0149
Scenario 2: Exclusion of Medication Users	Q1	390/2161					
	Q2		1.675(1.0497,2.6922)	0.5158	0.2399	4.6223	0.0316
	Q3		2.1603(1.3351,3.5268)	0.7703	0.2475	9.6882	0.0019
	Q4		1.8207(1.1221,2.9809)	0.5992	0.2489	5.7943	0.0161
Scenario 3: Exclusion of Lifestyle Modifications	Q1	422/2161					
	Q2		1.7075(1.0954,2.6791)	0.535	0.2279	5.5126	0.0189
	Q3		2.3512(1.4843,3.756)	0.8549	0.2366	13.0578	0.0003
	Q4		1.8807(1.1835,3.012)	0.6316	0.238	7.0401	0.008
Scenario 4: Exclusion of Any Hypertension Control	Q1	377/2161					
	Q2		1.6736(1.0388,2.7174)	0.515	0.2449	4.4206	0.0355
	Q3		2.1568(1.323,3.5497)	0.7686	0.2514	9.3446	0.0022
	Q4		1.8687(1.1432,3.0845)	0.6253	0.2529	6.1133	0.0134
Scenario 5: Exclusion of Control Behaviors	Q1	376/2161					
	Q2		1.678(1.0395,2.7301)	0.5176	0.246	4.4282	0.0354
	Q3		2.1709(1.3291,3.5802)	0.7751	0.2525	9.4262	0.0021
	Q4		1.8427(1.1243,3.0493)	0.6112	0.2542	5.7807	0.0162

Scenario 6: Exclusion of Other Interventions	Q1	662/1962					
	Q2		1.7095(1.0599,2.7762)	0.5362	0.2453	4.7769	0.0288
	Q3		2.3844(1.4702,3.9045)	0.8689	0.2489	12.1885	0.0005
	Q4		1.9071(1.1651,3.1495)	0.6456	0.2534	6.4902	0.0108
Scenario 7: Exclusion of Lifestyle-Only Management	Q1	510/1962					
	Q2		1.549(1.0172,2.3698)	0.4376	0.2155	4.1219	0.0423
	Q3		2.4254(1.5769,3.7589)	0.886	0.2214	16.0144	0.0001
	Q4		1.7538(1.1313,2.7362)	0.5618	0.2251	6.2292	0.0126
**Dimension:HBS**							
Original Model	Q1	821/2161					
	Q2		1.0254(0.7077,1.4865)	0.0251	0.1892	0.0175	0.8946
	Q3		1.2002(0.8453,1.7079)	0.1825	0.1793	1.0362	0.3087
	Q4		0.9405(0.6545,1.3528)	-0.0613	0.1851	0.1097	0.7404
Scenario 1: Exclusion of Prior Diagnosis	Q1	294/2161					
	Q2		1.1274(0.6797,1.8778)	0.1199	0.2587	0.2147	0.6431
	Q3		1.2829(0.7925,2.0937)	0.2491	0.2474	1.014	0.3139
	Q4		1.1003(0.6769,1.8004)	0.0955	0.2491	0.1471	0.7013
Scenario 2: Exclusion of Medication Users	Q1	390/2161					
	Q2		0.8575(0.5469,1.3433)	-0.1538	0.2289	0.4514	0.5017
	Q3		1.0622(0.697,1.6244)	0.0604	0.2156	0.0784	0.7795
	Q4		0.9508(0.6221,1.4571)	-0.0504	0.2168	0.0541	0.8161
Scenario 3: Exclusion of Lifestyle Modifications	Q1	422/2161					
	Q2		0.9316(0.6056,1.4337)	-0.0708	0.2196	0.104	0.7471
	Q3		1.256(0.838,1.891)	0.2279	0.2074	1.2082	0.2717
	Q4		1.0904(0.7261,1.6433)	0.0866	0.2081	0.173	0.6774
Scenario 4: Exclusion of Any Hypertension Control	Q1	377/2161					
	Q2		0.9255(0.585,1.4649)	-0.0775	0.2338	0.1098	0.7404
	Q3		1.1482(0.746,1.7758)	0.1382	0.2209	0.3915	0.5315

	Q4		1.0504(0.6817,1.6254)	0.0492	0.2214	0.0494	0.8242
Scenario 5: Exclusion of Control Behaviors	Q1	376/2161					
	Q2		0.9243(0.5833,1.4656)	-0.0787	0.2347	0.1125	0.7374
	Q3		1.1231(0.728,1.7407)	0.1161	0.2221	0.2734	0.601
	Q4		1.0475(0.6787,1.6235)	0.0464	0.2222	0.0436	0.8345
Scenario 6: Exclusion of Other Interventions	Q1	662/1962					
	Q2		1.0855(0.6905,1.7099)	0.082	0.2311	0.126	0.7226
	Q3		1.1587(0.7495,1.7982)	0.1473	0.223	0.4366	0.5087
	Q4		1.2824(0.8298,1.9916)	0.2488	0.2231	1.2436	0.2648
Scenario 7: Exclusion of Lifestyle-Only Management	Q1	510/1962					
	Q2		1.0367(0.6911,1.5566)	0.0361	0.2069	0.0304	0.8617
	Q3		1.2631(0.8619,1.8579)	0.2335	0.1958	1.4234	0.2328
	Q4		1.0622(0.7207,1.5695)	0.0603	0.1984	0.0925	0.7611
**Dimension:LBS**							
Original Model	Q1	821/2161					
	Q2		1.4645(0.9902,2.1726)	0.3815	0.2003	3.6281	0.0568
	Q3		1.5628(1.0411,2.3547)	0.4465	0.2081	4.6044	0.0319
	Q4		1.8826(1.2378,2.8764)	0.6327	0.215	8.6615	0.0032
Scenario 1: Exclusion of Prior Diagnosis	Q1	294/2161					
	Q2		0.987(0.5736,1.7017)	-0.0131	0.2769	0.0022	0.9623
	Q3		1.0385(0.5987,1.8096)	0.0377	0.2817	0.0179	0.8934
	Q4		1.7593(1.0212,3.0633)	0.5649	0.2799	4.074	0.0435
Scenario 2: Exclusion of Medication Users	Q1	390/2161					
	Q2		1.3087(0.8086,2.1282)	0.269	0.2465	1.1909	0.2751
	Q3		1.4212(0.8675,2.3441)	0.3515	0.2533	1.9263	0.1652
	Q4		1.8675(1.1313,3.1099)	0.6246	0.2577	5.8748	0.0154
Scenario 3: Exclusion of Lifestyle Modifications	Q1	422/2161					
	Q2		1.4604(0.9226,2.3234)	0.3787	0.2353	2.5898	0.1076

	Q3		1.6159(1.0116,2.5988)	0.4799	0.2405	3.9828	0.046
	Q4		1.8737(1.158,3.0558)	0.6279	0.2473	6.4459	0.0111
Scenario 4: Exclusion of Any Hypertension Control	Q1	377/2161					
	Q2		1.2347(0.7562,2.0246)	0.2108	0.2508	0.7063	0.4007
	Q3		1.3229(0.8019,2.1963)	0.2798	0.2567	1.1882	0.2757
	Q4		1.8194(1.0968,3.0454)	0.5985	0.2602	5.29	0.0214
Scenario 5: Exclusion of Control Behaviors	Q1	376/2161					
	Q2		1.2348(0.7549,2.0288)	0.2109	0.2518	0.7016	0.4023
	Q3		1.3247(0.8013,2.2044)	0.2812	0.2578	1.1898	0.2754
	Q4		1.7995(1.0818,3.0204)	0.5875	0.2616	5.0422	0.0247
Scenario 6: Exclusion of Other Interventions	Q1	662/1962					
	Q2		1.1403(0.7018,1.8593)	0.1313	0.2483	0.2795	0.597
	Q3		1.344(0.8175,2.2225)	0.2957	0.2549	1.3459	0.246
	Q4		1.6594(1.0051,2.7602)	0.5064	0.2575	3.8687	0.0492
Scenario 7: Exclusion of Lifestyle-Only Management	Q1	510/1962					
	Q2		1.4121(0.9164,2.1846)	0.345	0.2214	2.4292	0.1191
	Q3		1.5772(1.0123,2.4706)	0.4556	0.2274	4.0149	0.0451
	Q4		1.866(1.1829,2.9644)	0.6238	0.2342	7.0961	0.0077

***Original Model: The primary model based on the full study population, without exclusion of participants with potential bias.**
